# Grafting the surface of carbon nanotubes and carbon black with the chemical properties of hyperbranched polyamines

**DOI:** 10.1080/14686996.2016.1221728

**Published:** 2016-09-12

**Authors:** Francisco Morales-Lara, María Domingo-García, Rafael López-Garzón, María Luz Godino-Salido, Antonio Peñas-Sanjuán, F. Javier López-Garzón, Manuel Pérez-Mendoza, Manuel Melguizo

**Affiliations:** ^a^ Dpto. de Química Inorgánica, Facultad de Ciencias, Universidad de Granada, Granada, Spain; ^b^ Dpto. de Química Inorgánica y Orgánica, Facultad de Ciencias Experimentales, Universidad de Jaén, Jaén, Spain; ^c^ Andaltec, Plastic Technology Center, Jaén, Spain

**Keywords:** Carbon nanomaterials, functionalization, hyperbranched polyamines, 10 Engineering and Structural materials, 104 Carbon and related materials, 105 Low-Dimension (1D/2D) materials, 212 Surface and interfaces, 301 Chemical syntheses / processing, 502 Electron spectroscopy

## Abstract

Controlling the chemistry on the surface of new carbon materials is a key factor to widen the range of their applicability. In this paper we show a grafting methodology of polyalkylamines to the surface of carbon nanomaterials, in particular, carbon nanotubes and a carbon black. The aim of this work is to reach large degrees of covalent functionalization with hyperbranched polyethyleneimines (HBPEIs) and to efficiently preserve the strong chelating properties of the HBPEIs when they are fixed to the surface of these carbon materials. This functionalization opens new possibilities of using these carbon nanotubes-based hybrids. The results show that the HBPEIs are covalently attached to the carbon materials, forming hybrids. These hybrids emerge from the reaction of amine functions of the HBPEIs with carbonyls and carboxylic anhydrides of the carbon surface which become imine and imide bonds. Thus, due to the nature of these bonds, the pre-oxidized samples with relevant number of C=O groups showed an increase in the degree of functionalization with the HBPEIs. Furthermore, both the acid-base properties and the coordination capacity for metal ions of the hybrids are equivalent to that of the free HBPEIs in solution. This means that the chemical characteristics of the HBPEIs have been efficiently transferred to the hybrids. To reach this conclusion we have developed a novel procedure to assess the acid-base and the coordination properties of the hybrids (solids) by means of potentiometric titration. The good agreement of the values obtained for the hybrids and for the free HBPEIs in aqueous solution supports the reliability of the procedure. Moreover, the high capacity of the hybrids to capture Ni^2+^ by complexation opens new possibilities of using these hybrids to capture high-value metal ions such as Pd^2+^ and Pt^2+^.

## Graphical abstract

**Figure:**
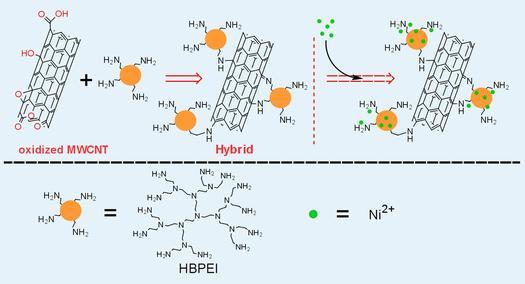

Hyperbranched polyethylenimines are covalently fixed to the surface of carbon materials, so the chemical characteristics of the amines are transferred to the hybrid materials.

## 1. Introduction

Grafting chemical functions to carbon materials allows combination of the specific physical and physicochemical properties of carbon materials with those of the bound chemical functions.[1–8] The synergy of combining these substances provides additional features and widens their potential applications. There are two general procedures for the functionalization of carbon materials: covalent and non-covalent. The latter usually relies on weak interactions (such as wrapping with large molecules, π–π interactions or deposition) between the host carbon material and the guest molecule.[9–12] The strength of the bond between the carbon material and the guest function is the main advantage of covalent functionalization.[13] The main drawback is that the covalent functionalization can change the structure of the support to some extent, altering the conjugated bonds of the graphenic layers and, thus, affecting some of their properties. In particular, the electronic and optoelectronic characteristics may be altered,[14–20] which might be crucial in carbon nanotubes and graphene based materials. That said, the stability of the bond achieved by covalent functionalization of the carbon material with the guest function is essential for many applications.[1,4,13,21–25]

Several procedures for the functionalization of carbon based materials throughout amine functions have been reported.[22,26–35] One option is to generate a primary functionalization with electrophilic groups which behave as anchors for the amine functions, due to their nucleophilic character. The first stage of most of these procedures consists in the treatment of the carbon materials with strong oxidants which result in the covalent attachment of several oxygen containing groups (cf. Figure S1 of Supplementary Data [SD]). The analysis of the structure and reactivity of these oxygen containing groups points out that not all of them are able to react with amine groups (a summary of the reactivity is shown in Table S1 of SD). This is the case of ethers, phenols, hydroxyls, epoxides and lactones which do not react with amine groups under mild conditions. Indeed, the aminolysis of epoxides in organic molecules is known to proceed following an SN2 or a borderline SN2-electrophillically assisted mechanism depending on the experimental conditions. But in these cases the aminolysis is only compatible with an attack of the nucleophilic amine to the carbon epoxide following a trajectory that forms an angle of *c*.180 ° with the C–O bond of the epoxide. Such an attack of the amine to the epoxide ‘from the back’ of the C–O bond is not possible for epoxide functions on the surface of the carbon materials due to stereochemical restraints. Nevertheless, other chemical functions such as cyclic anhydrides (of five and six members), quinones and carbonyls do react with the amine groups. Similarly, carboxylic acids are able to react with amines through acid-base reaction, resulting in the formation of ammonium carboxylates, which are not useful for our purposes. To avoid this, the carboxylic acids are usually transformed into acyl chlorides [22,23] by the reaction with inorganic acid chlorides, typically thionyl chloride SOCl_2_. The high electrophilic character of the acyl chlorides allows them to easily attack the primary and secondary amine groups to render secondary and tertiary amides, respectively. Thus, these amide groups are responsible of the covalent attachment of the molecule to the carbon material. Nevertheless, there is an easier procedure which consists in the transformation of the carboxylic groups into methyl esters, which can be further used to fix polyamines on carbon materials through amide bonds.[36–38]

The aim of this work is the covalent functionalization of multi-walled carbon nanotubes (MWCNTs) and a carbon black with polymeric amines. Two hyperbranched polyethyleneimines (HBPEI) of average molecular weight M_n_ = 600 and 1800 gmol^−1^ have been selected for this purpose. HBPEIs are water-soluble, nitrogen-rich homopolymers that possess a large proportion of terminal, primary amines (41 and 38% for the selected amines, respectively), which are needed to produce stable bonds by reaction with some oxygen functional groups on the surface of carbon-based materials. These include cyclic anhydrides and carbonyls that do not form stable linkages by reaction with secondary or tertiary amines. In that respect, the hyperbranched isomers of polyethyleneimines (HBPEIs) are more suitable than the linear polyethyleneimines (LPEIs, with only two terminal amino groups) to give hybrid materials in which the polyamine is linked to the carbon surface through several bonds. This shall finally result in a larger stability of these HBPEIs/hybrid materials. Moreover, HBPEIs are ideal for chelate formation due to their structure in which consecutive nitrogen bonds are always in 1,4 relative position. In addition, the repetition of these chelating units in the structure of the polyamines means that both molecules are able to form chelates with more than one metal ion per molecule, i.e. they can behave as poly-chelatogens.[38] Also regarding their chelating properties, it is known that branched PEIs form more stable metal complexes than LPEIs due to entropic factors.[39] Thus, the covalent attachment of these polyamines to the surface of carbon materials can result in stable hybrid materials which can capture relevant amounts of metal ions by coordination given that the properties of the polyamines are preserved in the hybrids. Therefore, an important goal of this work is to check if the hybrid materials keep the chelating property and other chemical characteristics of the polyamines. The former property, in turn, is the tool to cover the surface of the carbon-based materials with transition metal atoms in the form of complexes.

## 2. Experimental details

The products obtained by the partial oxidation of commercial multi-walled carbon nanotubes (Nanocyl 3100) and carbon black (CSX-691, from Cabot Corp., Boston, MA, USA) have been used. The former is labeled MW and the latter CSX. The surface area of both samples has been obtained by applying the Brunauer-Emmett-Teller (BET) equation to the nitrogen adsorption isotherms. The isotherms were obtained at 77 K by using ASAP 2020 equipment. The methodologies for oxidation and characterization of the samples have been already reported [40].

Samples MW/pH 9 and CSX/pH 9 were obtained by flowing ozone for 1 h in an aqueous suspension (at pH 9 and 25 °C) of MW and CSX. In addition, a treatment of the parent carbon materials with ozone gas for 60 min results in samples MW/O3G(60) and CSX/O3G(60). Four more samples were prepared by the reaction of MW and CSX with oxygen plasma for 2 min (samples MW/OP(2) and CSX/OP(2)), 10 min (sample CSX/OP(10)) and 30 min (sample MW/OP(30)). The aim supporting the use of these oxidized samples is to take advantage of the oxygen containing groups to act as intermediate for the further functionalization with HBPEIs.

**Figure 1.**
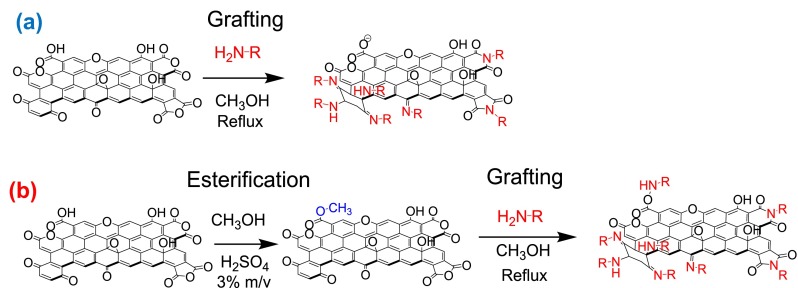
The two procedures of functionalizing oxidized carbon materials with HBPEI: (a) direct grafting and (b) through esterification.

Some of the oxidized samples were subjected to esterification [36,38] prior to the reaction with the HBPEIs. For this purpose, 1 g of oxidized sample was treated with 50 ml of a 3% (w/v) sulfuric acid in methanol solution. The suspension was refluxed in an inert atmosphere for 24 h. As already mentioned, this procedure is an easy alternative to the formation of acyl chlorides. The term ‘Est’ has been added to the name of these esterified samples. Figure 1 shows the two procedures used for the functionalization of the oxidized carbon materials with the HBPEIs. All these samples have been functionalized with a HBPEI of mean molecular weight M_n_=1800 g mol^−1^ (HBPEI_1800_). In addition, a HBPEI of M_n_= 600 g mol^−1^ (HBPEI_600_) was also used to functionalize some oxidized MW.

Both HBPEIs are commercially available from Sigma Aldrich (St. Louis, MO, USA). HBPEI_1800_ has been purified by azeotropic distillation with toluene as it is marketed as an aqueous solution (50/50 w/w) while HBPEI_600_ has been used without further purification. The characterization of both amines has been carried out by ^13^C nuclear magnetic resonance (NMR), thermogravimetric analysis (TGA) and acid-base titrations.

Bruker advance 400 (Bruker, Billerica, MA, USA) and Mettler Toledo (Greifensee, Switzerland) mod TGA/SDTA 851 instruments were used to obtain the NMR spectra and the gravimetric plots, respectively. The acid-base characteristics have been determined by potentiometric measurements. The experimental set-up for this purpose consisted in an automatic device equipped with a METROHM glass-calomel electrode (titration electrode), a Metrohm 765 Dosimat autoburete (0.001 ml dose accuracy), a Metrohm 713 pH-meter control unit (Herisau, Switzerland) and a Selecta Frigiterm 6000382 thermostatic bath. The equipment is controlled by a computer which uses Pasat software [41] for the data acquisition. The titrations of the HBPEI/H^+^ solutions were carried out at a molar concentration of 6 × 10^−3^ M. N(CH_3_)_4_Cl was used as electrolyte to fix the ionic strength to 0.1 M in chlorides. The solutions were continuously stirred, kept at 25 °C and in nitrogen atmosphere. The starting pH was set up at 2.5 by adding 0.1 M HCl solution. The titrating agent was N(CH_3_)_4_OH which was consecutively added in volumes of 30 μl. Moreover, the coordination capacity of both polyamines for Ni^2+^ has been studied in the same experimental set-up used for the acid-base titrations.

The carbon materials were suspended in methanol solutions of HBPEI for 24 h for the functionalization with the polyamines. The suspensions were heated under reflux in the case of MW and at 70 °C in a sealed tube in the case of CSX. The carbon-material/HBPEI/methanol ratio was 1/2/50 (w/w/v). Typically, batches of 100 mg of MW or CSX were used. This procedure allowed carbon materials/HBPEI hybrids to be obtained. The label of these hybrid materials contains the name of the parent oxidized sample to which 1800 (for HBPEI_1800_) or 600 (for HBPEI_600_) has been appended. For instance, the name MW/OP(30)-Est-1800 refers to a hybrid material based on carbon nanotubes which has been obtained by the reaction of the HBPEI_1800_ with the oxidized (by oxygen plasma for 30 min) and esterified carbon material.

The characterization of the hybrid materials has been carried out by using several techniques. Elemental analysis (EA) of the hybrids has been performed with a Thermo Finnigan Flash EA1112 instrument (Thermo Fisher Scientific, Waltham, MA, USA). X-ray photoelectron spectroscopy (XPS) measurements were performed with a Kratos Axis Ultra DLD spectrometer (Kratos, Manchester, UK). Monochromatic Al/MgK_α_ radiation from twin anode in constant analyzer energy mode with pass energy of 160 and 20 eV (for the survey and high resolution spectra, respectively) was used. The C 1s transition at 284.6 eV was used as reference to obtain the binding energies of heteroatoms. The FTIR and Raman spectra have been recorded with Jasco FP-6200 and Jasco NRS-5100 equipment (Jasco, Easton, MD, USA), respectively. An important aspect of this work is to know whether the properties of the HBPEIs are preserved in the carbon materials/HBPEI hybrids. This has been analyzed by comparing the acid-base characteristics and the coordination capacity for Ni^2+^ of both free polyamines in solution with these of the hybrids. In addition, the surface charge density (Q in meq H^+^/g of adsorbent) of both original materials and some selected hybrids was determined by potentiometric titration measurements. Thus, Q was calculated by means of the equation Q = 1/m(V_o_+V_t_) ([H]_i_-[OH]_i_-[H]_e_+[OH]_e_), where V_o_ and V are the initial solution and titrant volumes, respectively, and m is the mass of the adsorbent. Subscripts i and e refer to the initial and the equilibrium concentration of protons or hydroxyl ions. Then, the proton isotherms are obtained by plotting Q vs. equilibrium pH values.

If the chelating capacity of the polyamines is preserved in the hybrids, they must present a larger capacity to capture Ni^2+^ from aqueous solutions than the parent carbon materials. Thus, the Ni^2+^ retention isotherms of several hybrids and of the parent carbon materials have been also obtained. For this purpose, 25 mg of hybrid material were added to several solutions of Ni^2+^ (0.05×10^−1^–3×10^-1^ M). The ionic strength of the solution was kept constant (0.1 M), the temperature was 25 °C, the contact time 24 h and the pH was set to 6. The retention isotherms were obtained by measuring the Ni^2+^ concentration at the equilibrium by inductively coupled plasma mass spectrometry (ICP-MS). Moreover, high-resolution transmission electron microscopy (HRTEM) micrographs and energy-dispersive X-ray spectroscopy (EDX) of some selected hybrid materials containing Ni^2+^ have been obtained. An ultrahigh resolution electron microscope FEI Titan G2 was used for this purpose.

## 3. Results and discussion

As previously mentioned, the structural characteristics of the HBPEIs have been determined. Both polyamines are statistical polymers obtained by ring-opening polymerization of aziridine in acid solution.[42,43] Therefore, their degrees of branching (DB) as well as their polydispersity index (PD) are relevant parameters. The amounts of primary, secondary and tertiary amines are needed to determine the DB.[44] These parameters have been obtained from the quantitative ^13^C-NMR spectra [45–48] (cf. SD) and the results of the characterization of both polymers are shown in Table 1. Regarding these data, it is worth noting that the degree of branching presents intermediate values (64–65 %), so the ‘internal’ amine groups are highly accessible, which can condition the chemical behavior of the carbon materials/HBPEI hybrids.

**Table 1.** Characteristics of the HBPEIs.

		HBPEI_600_	HBPEI_1800_
	M_n_ (g·mol^−1^)	600	1800
	PD	14.0	41.8
	DB (%)	65	64
	Primary	41 (6)^a^	35 (15)^a^
Amines (%)	Secondary	35 (5)^a^	36 (15)^a^
	Tertiary	24 (3)^a^	29 (12)^a^

^a^ Number of nitrogen atoms of this amine group in the model molecule.

**Figure 2.**
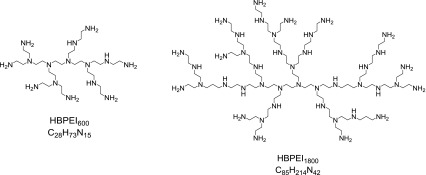
Molecular structure of both hyperbranched polyethyleneimines.

According to these data, the drawings in Figure 2 are close representations of prototype molecules of both polyamines.

The thermal decomposition profiles of both polyamines are very similar (cf. SD). The largest difference between both thermograms is the relative important weight loss (near 14%) of the HBPEI_600_ in the range 100–165 °C, which is due to the water content of this polyamine. The temperature range for the dehydration process is coherent with the strong retention of water by the HBPEI through hydrogen bonds. Apart from this, both thermograms are similar and have an important weight loss in a relative narrow temperature range (200–400 °C). The final residual mass is only 5 and 1% of the initial mass of HBPEI_600_ and HBPEI_1800_ respectively, which indeed accounts for the nearly total decomposition of both polymers.

The results of the functionalization of the oxidized carbon materials with HBPEI_600_ and HBPEI_1800_ are shown in Table 2. The HBPEI contents have been determined from EA and TGA measurements. The chemical composition of the materials obtained by EA is summarized in SD (Table S2). Thus, the nitrogen content measured by the former technique allows the polyamine in the hybrid materials to be determined, assuming that the HBPEIs are homo-polymers with the elemental composition of ethyleneimine (or aziridine). The thermograms of the hybrids are also shown in SD (Figures S5 and S6).

**Table 2.** Amount of HBPEI fixed on the oxidized carbon materials.

Sample	% HBPEI (EA)	% HBPEI (TGA)	Surface density of HBPEI (mg m^−2^)
MW/pH 9-1800	7.9	5.7	0.36
MW/O3G(60)-1800	11.3	8.2	0.53
MW/OP(2)-1800	8.5	7.4	0.39
MW/OP(30)-1800	11.9	11.5	0.57
MW/pH 9-Est-1800	8.2	6.0	0.37
MW/OP(2)-Est-1800	8.3	6.8	0.38
MW/OP(30)-Est-1800	10.0	9.6	0.46
MW/pH 9-600	4.8	4.1	0.21
MW/OP(30)-600	8.4	7.2	0.38
MW/pH 9-Est-600	5.3	3.4	0.23
CSX/pH 9-1800	1.8	1.6	0.93
CSX/O3G(60)-1800	2.5	2.0	1.28
CSX/OP(2)-1800	2.1	1.7	1.05
CSX/OP(10)-1800	2.4	2.2	1.25
CSX/pH 9-Est-1800	1.7	1.6	0.86
CSX/O3G(60)-Est-1800	1.9	1.6	0.95
CSX/OP(2)-Est-1800	1.8	1.4	0.94
CSX/OP(10)-Est-1800	2.0	1.9	1.03

The data in Table 2 also show that the amounts of polyamine fixed on MW are much larger than these on CSX. This is due to the former has a larger surface area (240 m^2^ g^−1^) than the CSX (20 m^2^ g^−1^). These values have been obtained by fitting the nitrogen adsorption data (SD) to the BET equation. Moreover, the defects, irregularities and connection nodes in MW are active sites that can also react with the amines by addition to double bonds.[49–54] So, if the amounts of fixed polyamines are normalized to the surface area, the values range between 0.35–0.57 mg m^−2^ in the MW-series and 0.64–1.25 mg m^−2^ in the CSX-series. This is in agreement with the expected larger reactivity of the carbon black due to the higher number of defects present in this material. In relation to the HBPEI_600_, the amount of this polyamine fixed to the nanotubes is clearly smaller than this of HBPEI_1800_. This is the expected trend, as the mass fixed by the HBPEI_600_ has to be necessarily smaller than this of the HBPEI_1800_ for the same number of covalent bounds between the amine and the MW.[27] Thus, it is evident that the degree of functionalization that can be attained with the HBPEI_600_ is clearly smaller than with HBPEI_1800_.

The degrees of functionalization with the polyamines of the esterified samples are similar to or smaller than those of the non-esterified carbon materials. This suggests a partial elimination of the oxygen containing groups of the oxidized samples under the experimental conditions of the esterification. The data in Table 2 also show that the largest degrees of functionalization with polyamine are reached in the samples oxidized by oxygen plasma treatments, i.e. this is the most efficient oxidation procedure for the further functionalization with HBPEI. This is probably related to the largest amount of oxygen containing groups reached by the plasma treatment.[40]

Evidence of the strong interaction between the HBPEIs and the carbon materials is that the hybrids were thoroughly washed (with methanol under reflux in a Soxhlet extractor for 24 h in the case of the MW/hybrids and five times at 70 °C for 30 min in the case of CSX/ hybrids) after the reactions and before being analyzed. It is worth noting that both polyamines are highly soluble in methanol so the fraction of physically adsorbed polyamines has to be necessarily eliminated through the washing step.

**Figure 3.**
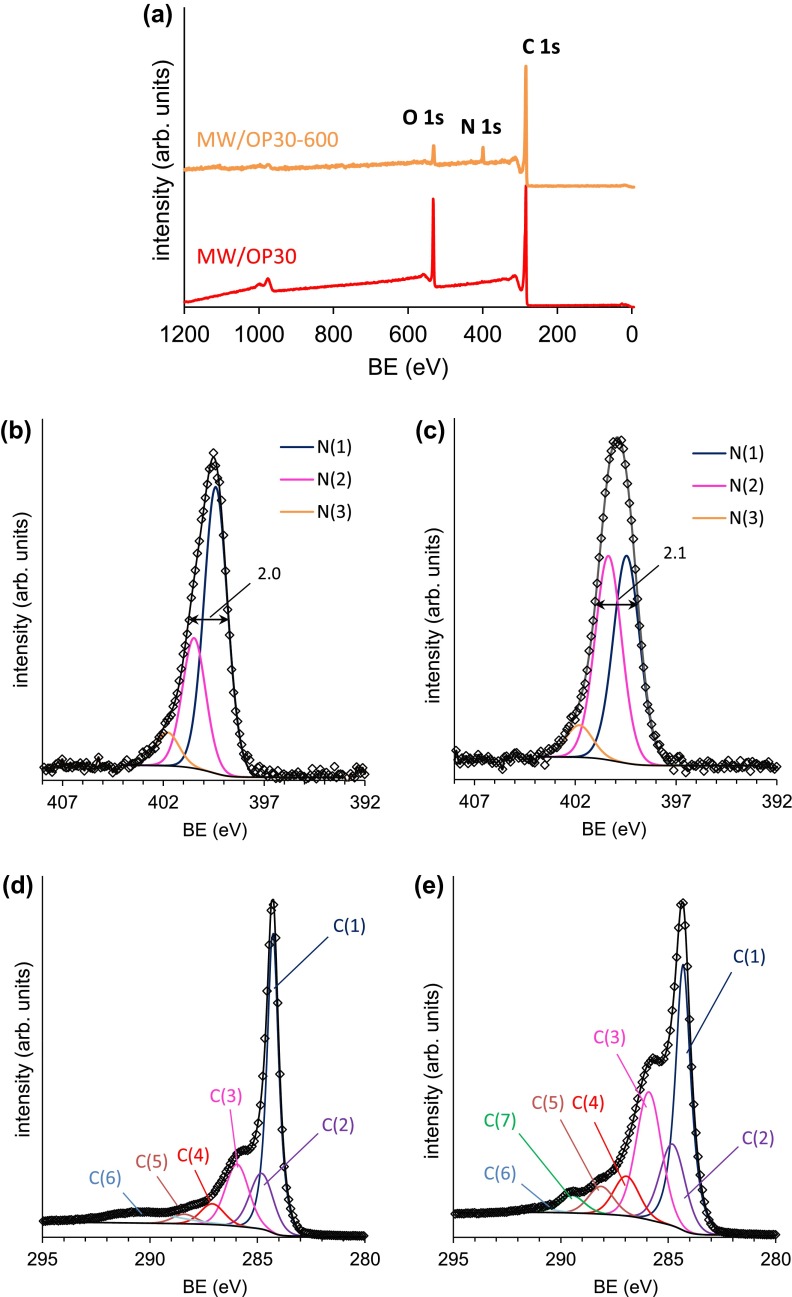
(a) Some selected XPS survey spectra; (b, c) deconvolution of high resolution N1s spectra of MW/OP(30)-1800 and CSX/O3G(60)-1800; (d, e) deconvolution of high resolution C 1s spectra of MW/OP(30)-1800 and CSX/OP(10)-1800. BE stands for binding energy.

Thus, the nitrogen peak at 400 eV in all of the survey XPS spectra of the hybrid materials (Figure 3(a)) is due to the covalent attachment of the HBPEI_600_ to the carbon material.

Further insight into the bonding of the polyamine molecule to the carbon materials has been obtained by analyzing the N 1s and C 1s spectra (Figure 3) obtained at higher resolution (20 eV pass energy). Figure 3(b) and 3(c) show high-resolution N 1s spectra of some selected samples. The spectra of the free HBPEIs have been already reported.[38,55] All these spectra of the hybrids contain an asymmetric peak at 399.4 eV. It can be deconvoluted into symmetric peaks which are described by Gaussian–Lorentzian functions. The fitting of the data has been carried out considering these peaks have the same full width at half maximum, and a Shirley type background was subtracted from the experimental data before the fittings were performed. Thus, the deconvolution of these spectra results in three components, namely N(1), N(2) and N(3) at 399.4 ± 0.1, 400.5 ± 0.2 and 401.7 ± 0.1 eV, respectively. The N(1) component is the most abundant (63–75%) in all cases. The N(2) abundance is smaller although significant (19–29%) and this of N(3) is the smallest (between 5 and 9%).

**Figure 4.**
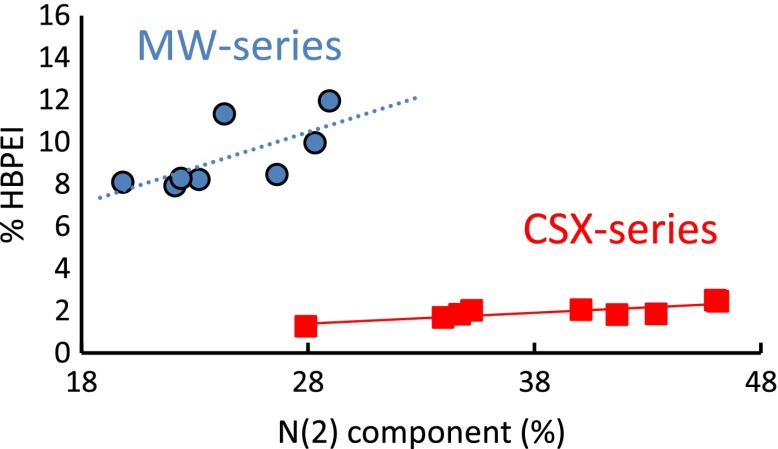
Amount of N(2) component vs. the HBPEI_1800_ content.

These three components can be assigned to amine, imine (or imide) and amide groups.[36,56] Indeed, the amine groups are the organic functions present in the HBPEIs. The imine, imide and amide groups can be the result of the reaction between the HBPEIs and the oxygen containing groups of the carbon materials. Nevertheless, these components could be also due to other factors such as intramolecular hydrogen bonds in the HBPEI fragments or to the presence of protonated nitrogen atoms (ammonium groups).[56–58] The signals of these factors overlap with the C-N functions of the covalent attachment of the polyamines to the carbon materials. Therefore, it is difficult to distinguish the individual contribution of these factors and of the several C-N groups in the N 1s peak. Nevertheless, Figure 4 shows an evident relationship between the amount of HBPEI_1800_ on the hybrids and the percentage of the N(2) component, suggesting that the HBPEI_1800_ is probably bound to both carbon materials through imine or imide bonds.

Additional information can be obtained from the C 1s XPS spectra (Figure 3). Figure 3(d) and 3(e) show some selected examples of the C 1s spectra obtained at high resolution. The main component of the C 1s spectrum is an asymmetric peak at 284.3 eV, labeled as C(1), which is assigned to graphitic C sp^2^. The main new feature of the C 1s spectra of the hybrid materials (both based on MW and CSX) is a very marked shoulder nearby the main peak. This shoulder appears as a very well-defined maximum in the samples having the largest amounts of HBPEI per surface area. This is the case of sample MW/OP(30)-1800 and also of the majority of hybrids of the CSX-series (Figure 3(d)).

The deconvolution of the C 1s peak results in six components (Table 3) which have been assigned according to the literature.[55,59–63] The one labeled C(3) (at 286.0 ± 0.1 eV) corresponds to sp^3^ carbon bound to nitrogen (-CH_2_-CH_2_-N<), which is the structural unit of the HBPEI. This component can also be assigned to C-N groups which have been formed by the already commented direct reaction of the polyamines with the C/C double bond of the carbon materials.

**Table 3.** Assignment of the C 1s components of the high resolution XPS spectra.

Component	BE (eV)	Assignment
C(1)	284.3 ± 0.1	Graphitic carbon
C(2)	284.9 ± 0.1	Non-conjugated carbon
C(3)	286.0 ± 0.1	C–O, as phenols or ethers, C sp^3^ bound to nitrogen (-CH_2_-CH_2_-N<)
C(4)	287.0 ± 0.2	C=O, of ketones and quinones, C=N, imines
C(5)	288.3 ± 0.2	N-C=O, carboxamide groups, O-C=O, carboxylic derivatives as carboxylic acids, ethers and anhydrides
C(6)	290.6 ± 0.4	Shake-up
C(7)	289.4 ± 0.1	-(O)C-N-C(O)-, carbons of imides taking part of 5 member cycles

In addition, this component can also be due to C–O groups of the oxidized materials. The C(4) component (at 287.1 ± 0.2 eV) can be assigned to imine groups (C=N) and carbonyl (C=O) of ketones and quinones. Other carbon-nitrogen groups cause the C(5) component (at 288.3 ± 0.2 eV) which is assigned to carboxamide (N-C–O) and also to carboxyl (O-C=O). The component at 290.6 ± 0.4 eV, C(6), is due to the π–π shake-up satellite. In the case of the CSX based hybrid materials there is an additional C(7) component at 289.4 ± 0.1 eV. This signal was not present in the oxidized precursors and can be attributed to functions containing carbon in a high oxidized state, such as urethane groups, though the formation of these groups from the oxygenated ones in the oxidized CSX precursors remains unclear.

**Figure 5.**
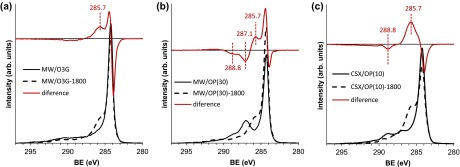
Examples of XPS difference spectra (see the text).

The comparison of the C 1s spectra of the oxidized samples to these of the hybrids allows further insight into the bonding between the HBPEIs and the carbon materials. The difference spectra in the C 1s region after normalization have been obtained with this aim. Figure 5 shows some selected examples. The wide XPS spectra of these samples are shown in SD. In Figure 5 a significant increase is seen in intensity of the component at 286 eV (Figure 5(a)) due to the incorporation of the HBPEI. Moreover, this peak is not symmetric but it has a tail extending to larger BE values. This tail can be associated to the C(4) component near 287 eV, which is compatible with imine groups (C=N). Thus, this fact supports the previous statement about the bonding of the HBPEI to the carbon materials through imine and imide groups.

The imine groups come out from the reaction between the carbonyl groups of the oxidized precursors and the primary amines of the HBPEI. Then, no neat increase of the signal is observed at a BE around 287 eV, since the imine C=N signal is formed at the expense of the close carbonyl C=O signal. Even more, the functionalization with HBPEI of the MW oxidized with oxygen plasma (Figure 5(b)) results in a decrease of this signal near 287 eV, due to the loss of labile groups, mostly C–O of hydroxyl and epoxy type, that take place upon treatment with methanol under the reaction conditions (cf. SD, Figure S9). An additional feature in the difference spectra is a small decrease of intensity in a relative wide range of B.E (288–290 eV) assigned to the most oxidized carbon atoms. This effect is more clearly seen in the CSX-series hybrid materials (Figure 5(c)) for which the decrease of signal appears at around 289 eV, and it is explained by a probable higher concentration of anhydrides per area with respect to the oxidized MWCNTs. Thus, these data support that the HBPEIs are covalently bound to the carbon materials through imine and imide groups.

**Figure 6.**
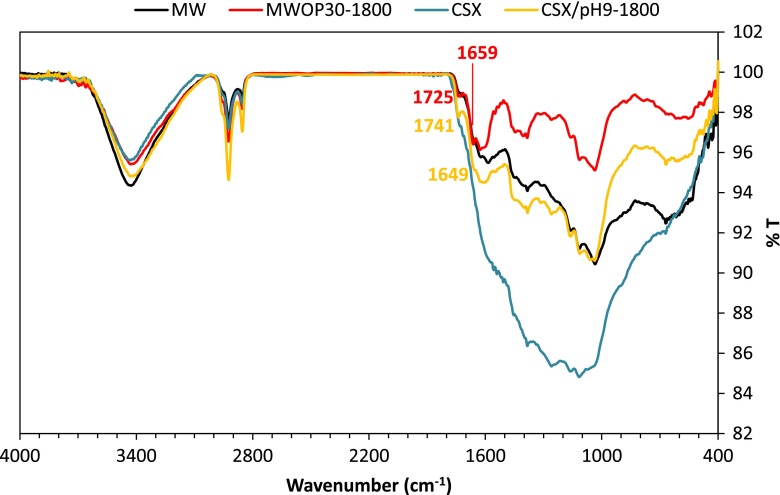
FTIR spectra of the original carbon nanotubes and of two hybrids.

Additional information on this issue can be obtained from the FTIR spectra. Figure 6 shows the spectra of the original carbon materials and two hybrids obtained from them.

It is seen that the hybrid materials show the characteristics peaks of the HBPEI. Unfortunately these peaks coincide with these of some oxygen containing groups. The HBPEI peaks include those at 3426 cm^−1^ (-N-H stretching), 2924 and 2856 cm^−1^(-C-H stretching), 1595 cm^−1^ (-N-H bending), 1418 cm^−1^ (-C-H bending) and several peaks in the range 1300–1000 cm^−1^ (-C-N stretching). We also detected two small peaks at 1725–1741 cm^−1^due to imide groups and at 1649–1659 cm^−1^ of the imine bonds, which support the covalent attachment of the HBPEIs.

**Figure 7.**
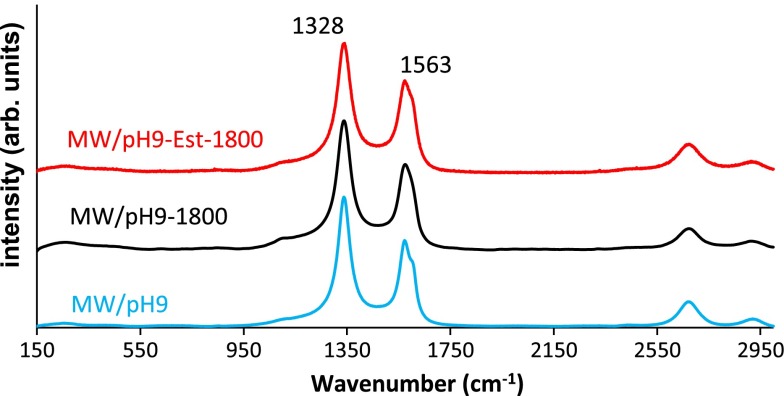
Raman spectra of oxidized carbon nanotubes and two hybrids.

The Raman spectra of some selected samples in Figure 7 show the typical bands of these materials. The first-order bands at 1328 and 1563 cm^−1^ are labeled as D and G. The first one is a disorder-induced peak while the second is due to sp^2^ ordered carbon networks. The I_D_/I_G_ intensity ratio is used to estimate the change in order-disorder of these materials. As it is seen in Figure 7 the spectra are very similar. Thus, the values of this ratio range between 1.36 and 1.41. This suggests that no new structural defects are produced in the graphitic structure as an outcome of the functionalization with the HBPEIs. This points out that the reaction of the polyamines is mainly produced with the previously fixed oxygen containing groups.

A very important issue of this work is to determine if the chelating properties and other characteristics of the HBPEIs are preserved after the covalent bonding to the carbon material surfaces, i.e. in the final hybrid materials. The acid-base behavior of the polyamines is a distinctive feature of these HBPEIs. For this reason, we have carried out acid-base titrations of the free polyamines and of the hybrids.

**Figure 8.**
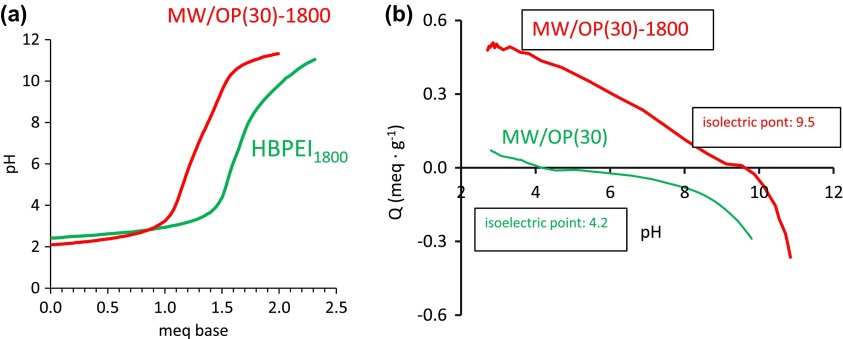
(a) Acid-base titration plots of the free HBPEI_1800_ and of one hybrid; (b) plots to obtain the zero point of charge.

Figure 8(a) shows the titration plots of the free HBPEI_1800_ in solution and one of selected hybrid materials. The titration profile of the hybrid is very similar to that of the polyamine. The small differences observed may be due to different accessibility to the amine groups in the free polyamines and in the hybrid. In addition, it may also be due to the remaining oxygen containing groups on the carbon material surfaces. Thus, the titration plots in this figure mean that the acid-base nature of the HBPEIs has been transferred to the hybrids. Moreover it is expected the surface charge of the hybrids is different to that of the oxidized carbon materials. With the aim of checking this fact we have carried out measurements of the isoelectric point of the oxidized and hybrid materials. Figure 8(b) shows the plots of an oxidized carbon nanotubes and of the hybrid containing the HBPEI_1800_. Similar plots of the CSX-series are in SD. It is seen that the oxidized material has an acid isoelectric point (4.2) due to the oxygen containing groups. Nevertheless the value of this point largely increases (9.5) in the hybrid due to the basic character of the HBPEI. Therefore, the plots also support that the characteristics of the polyamines have been transferred to the hybrids. An outcome of this transfer is that the hybrids are water dispersible (Figure S11).

**Figure 9.**
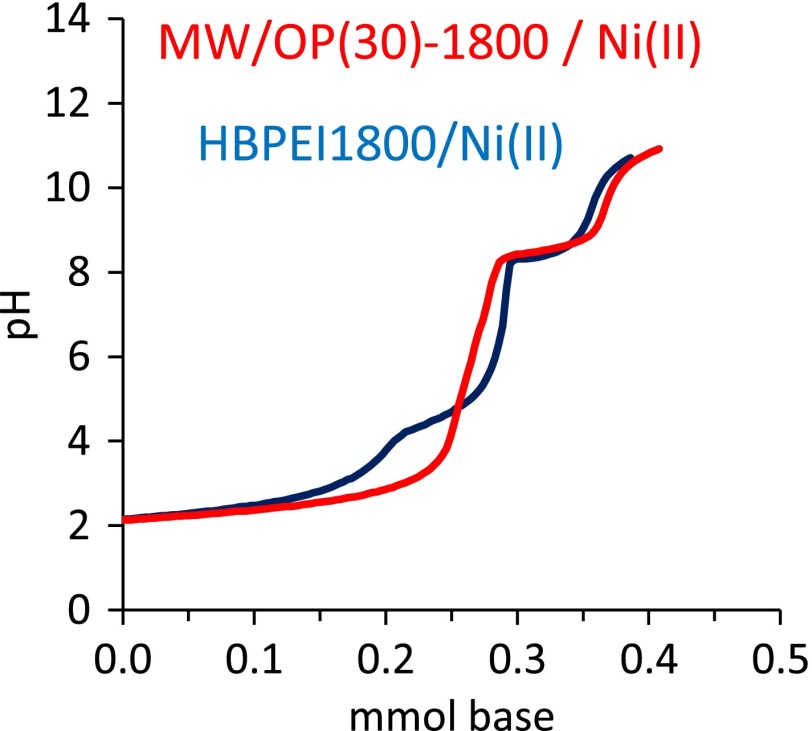
Ni^2+^ titration plots of the free HBPEI_1800_ (blue line) and of one hybrid (red line).

Other distinctive chemical characteristic of the HBPEIs is the complexing behavior for metal ions. This characteristic has been checked for both free HBPEIs in solution with Ni^2+^ by potentiometric titration (cf. experimental section). The plot obtained for HBPEI_1800_ is shown in Figure 9 (blue line) and the species distribution obtained from this plot (Figure S12) allows us to conclude that coordination compounds of Ni^2+^ with one, two or three amine groups acting as donor are formed in the reaction with both HBPEIs. The coordination equilibria and the values of the stability constants of these complexes are summarized in SD (Table S3). Interestingly the titration plot of the hybrid material with Ni^2+^ (red line in Figure 9) is very similar to that of the free polyamine. Although the titration of metal ions with complexing species in aqueous solution is quite common, as far as we know the titration of solid materials with metal ions in solution has not been reported. The fact that both plots in Figure 9 are similar means that the complexing properties of the HBPEIs have been transferred to the hybrids.

**Figure 10.**
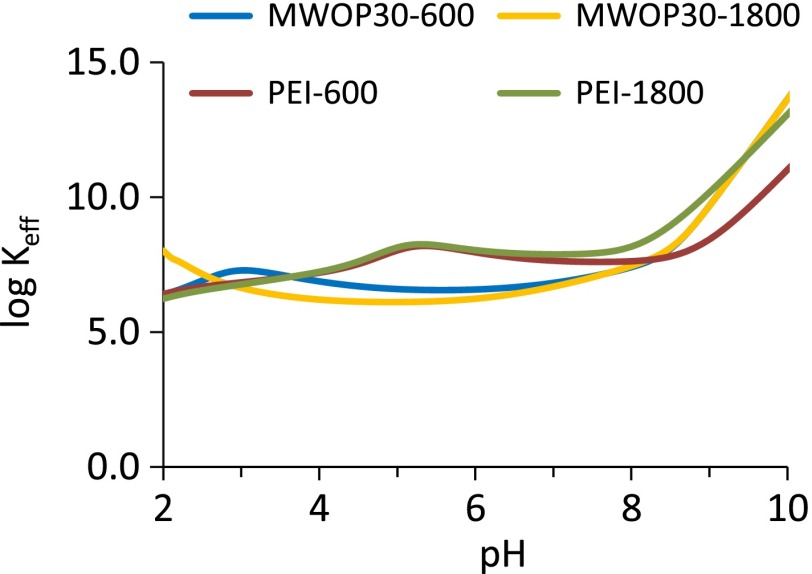
Variation of the effective complexation constants of the polyamines and of the hybrids with the pH.

**Figure 11.**
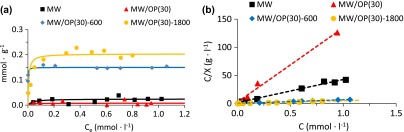
(a) Retention isotherms of Ni^2+^; (b) the fitting of the experimental data to the Langmuir equation.

Moreover, the variation of the effective complexation constants (cf. SD) of the free polyamines and of the hybrids with Ni^2+^ is similar (Figure 10). All these data support that the chemical characteristics of the HBPEIs have been transferred to the hybrids. Therefore, it is expected that the hybrid materials are able to capture larger amounts of Ni^2+^ by coordination at the interface than the parent oxidized carbon materials (Figure 11). The retention isotherms of two hybrid materials and of the pristine and oxidized carbon materials are presented in Figure 11(a), showing a large increase of the amount of Ni^2+^ captured by the hybrid materials. The fitting of these plots to the Langmuir equation (Figure 11(b)) also results in much larger values of Ni^2+^ captured by the hybrids (1.5·10^−4^ and 2·10^−4^ mol g^−1^) than by the parent carbon materials (0.1·10^−4^ and 0.2·10^−4^), which is an additional support for the successful transference of the complexing properties of the polyamines to the hybrid materials.

The reported amounts of Ni^2+^captured by carbon based materials range between 2 and 500 mg g^–1^ (Table S5 of SD and references therein). Thus the values reported in this work are near the lower end of this range of values, although they are close to many reported for carbon materials.

**Figure 12.**
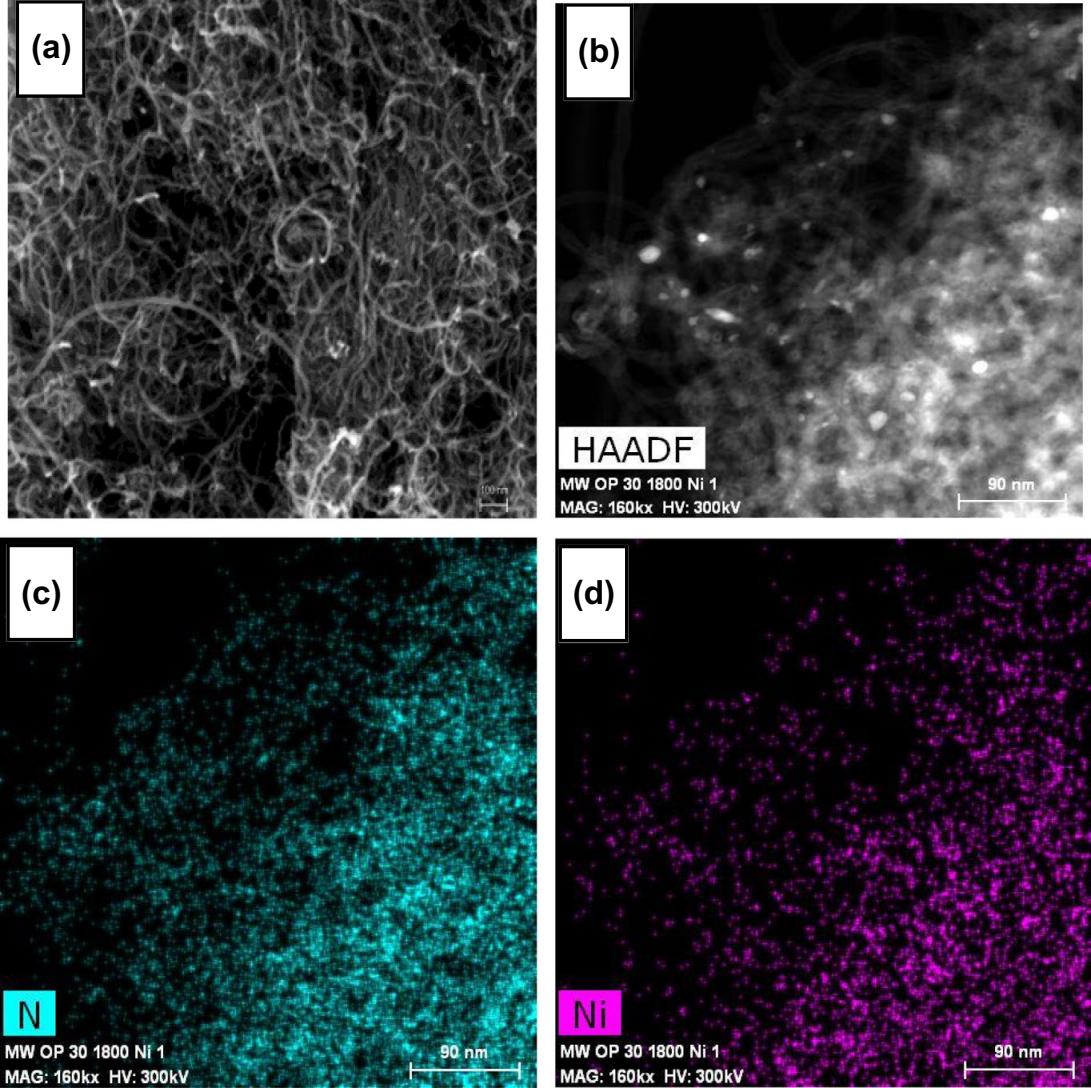
HRTEM images of: (a) MW/OP(30); (b) MW/OP(30)-1800/Ni; (c) map of nitrogen of sample MW/OP(30)-1800/Ni; (d) map of Ni of the same sample. HAADF stands for high-angle annular dark field.

Figure 12(a) and 12(b) show HRTEM images of an oxidized carbon nanotube material and of the hybrid. Similar images of the CSX-material are shown in SD. The comparison of the images of both series points out a larger degree of aggregation in the hybrids than in the original carbon materials. In addition Figure 12(b) reveals that the nanotubes are decorated with small spots of, presumably, Ni^2+^/HBPEI complexes. To check this hypothesis we have obtained the nickel and nitrogen maps of this image (Figure 12(c) and 12(d)) which clearly support the statement.

**Figure 13.**
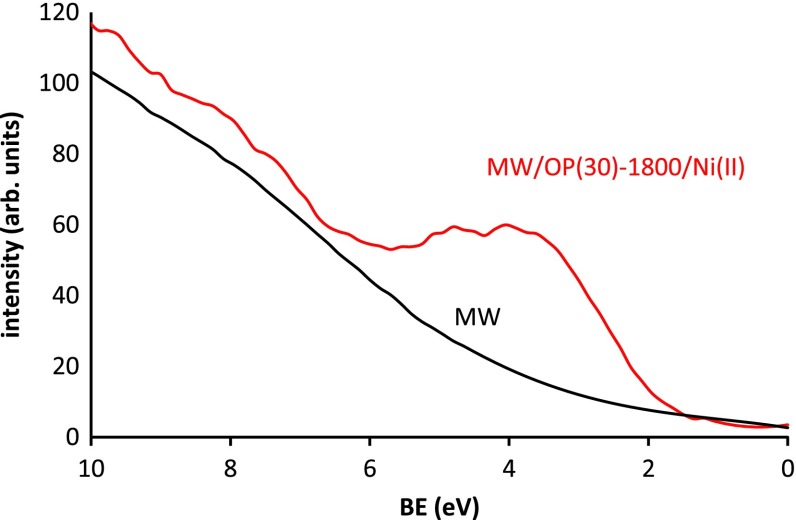
High resolution XPS spectra of the valence band region.

The electronic properties of the materials can be examined by means of the high resolution XPS spectra in the region of the binding energies near Fermi level. This region is very useful to reveal subtle differences in the electronic properties of solids in general and of carbon materials in particular. Figure 13 shows the spectra of the original carbon nanotubes and of a selected hybrid (MW/OP(30)-1800/Ni(II)). It is seen that the emission of photoelectrons of the original carbon nanotubes is almost negligible at binding energies smaller than 4–5 eV. Nevertheless this emission in the hybrid starts at smaller binding energy (near 2 eV) and has a relative maximum near 4 eV. This means that the electronic behavior of the hybrid has been altered in relation to the original nanotubes, which corresponds to a non-conductive material.

## 4. Conclusions

The level of functionalization on the surface of MWCNTs (MW) and a carbon black (CSX) with polyamines seems to be dependent on the oxygen content of these carbon materials. The largest degrees of functionalization are reached in the samples which have been oxidized by plasma treatment. Nevertheless, the oxidation with ozone allows similar degrees of functionalization of CSX with HBPEIs to be reached. In spite of this, the characteristics of the plasma process (very simple, fast and allows reactant saving) make this procedure ideal for further functionalization with HBPEIs. The esterification process does not improve the degree of functionalization, so it can be avoided. The fraction of HBPEI per mass unit attached to the CSX is smaller than that fixed to MW due to the smaller surface area of the former. The polyamines are covalently bound to the carbon materials by the formation of imine and imide groups. The chemical properties of the HBPEIs are transferred to the carbon material/HBPEI hybrids. This can allow these hybrid materials to be used in processes in which the HBPEIs are chemically active, for example, in recovering metal ions from solution by forming coordination compounds at the interface.

## Disclosure statement

No potential conflict of interest was reported by the authors.

## Funding

The financial support of the MINECO [projects MAT2014-60104-C2-1-R, MAT2014-60104-C2-2-R and CTM2010-16770], FEDER program, Autonomous Regional Government [Junta de Andalucía, Proyecto de Excelencia ref: P09-FQM-4765 and grupo RNM342] and Programa de Fortalecimiento de la I+D+I from UGR is acknowledged.

## Supplementary material

The supplementary material for this paper is available online at http://dx.doi.org/10.1080/14686996.2016.1221728

## Supplementary Material

Supplementary_data.pdf

## References

[CIT0001] Maiti UN, Lee WJ, Lee JM (2014). 25th anniversary article: chemically modified/doped carbon nanotubes & graphene for optimized nanostructures & nanodevices. Adv. Mater.

[CIT0002] Peng X, Wong SS (2009). Functional covalent chemistry of carbon nanotube surfaces. Adv. Mater.

[CIT0003] Hirsch A (2002). Functionalization of single-walled carbon nanotubes. Angew. Chemie Int. Ed.

[CIT0004] Balasubramanian K, Burghard M (2005). Chemically functionalized carbon nanotubes. Small.

[CIT0005] Singh P, Campidelli S, Giordani S (2009). Organic functionalisation and characterisation of single-walled carbon nanotubes. Chem. Soc. Rev.

[CIT0006] Li Z, Dai X, Du K (2016). Reduced Graphene Oxide/O-MWCNT hybrids functionalized with p-Phenylenediamine as high-performance MoS2 electrocatalyst support for hydrogen evolution reaction. J. Phys. Chem. C..

[CIT0007] Xing Z-C, Chang Y, Kang I-K (2010). Immobilization of biomolecules on the surface of inorganic nanoparticles for biomedical applications. Sci. Technol. Adv. Mater.

[CIT0008] Kamiya H, Iijima M (2010). Surface modification and characterization for dispersion stability of inorganic nanometer-scaled particles in liquid media. Sci. Technol. Adv. Mater.

[CIT0009] Fujigaya T, Nakashima N (2015). Non-covalent polymer wrapping of carbon nanotubes and the role of wrapped polymers as functional dispersants. Sci. Technol. Adv. Mater.

[CIT0010] Monthioux M (2012). Carbon meta-nanotubes.

[CIT0011] Krueger A (2010). Carbon materials and nanotechnology.

[CIT0012] Nakashima N (2006). Solubilization of single-walled carbon nanotubes with condensed aromatic compounds. Sci. Technol. Adv. Mater.

[CIT0013] Misak HE, Asmatulu R, O’Malley M (2014). Functionalization of carbon nanotube yarn by acid treatment. Int. J. Smart Nano Mater.

[CIT0014] Adhikari PD, Jeon S, Cha M-J (2014). Immobilization of carbon nanotubes on functionalized graphene film grown by chemical vapor deposition and characterization of the hybrid material. Sci. Technol. Adv. Mater.

[CIT0015] Bekyarova E, Itkis ME, Ramesh P (2009). Chemical modification of epitaxial graphene: spontaneous grafting of aryl groups. J. Am. Chem. Soc.

[CIT0016] Some S, Kim J, Lee K (2012). Highly air-stable phosphorus-doped n-type graphene field-effect transistors. Adv. Mater.

[CIT0017] Hwang JO, Park JS, Choi DS (2012). Workfunction-tunable, N-doped reduced graphene transparent electrodes for high-performance polymer light-emitting diodes. ACS Nano.

[CIT0018] Hwang SK, Lee JM, Kim S (2012). Flexible multilevel resistive memory with controlled charge trap B- and N-doped carbon nanotubes. Nano Lett.

[CIT0019] Abdelkader VK, Domingo-García M, Melguizo M (2015). Covalent bromination of multi-walled carbon nanotubes by iodine bromide and cold plasma treatments. Carbon N. Y.

[CIT0020] Li Y, Shu H, Niu X (2015). Electronic and optical properties of edge-functionalized graphene quantum dots and the underlying mechanism. J. Phys. Chem. C.

[CIT0021] Van Hooijdonk E, Bittencourt C, Snyders R (2013). Functionalization of vertically aligned carbon nanotubes. Beilstein J. Nanotechnol.

[CIT0022] Karousis N, Tagmatarchis N, Tasis D (2010). Current progress on the chemical modification of carbon nanotubes. Chem. Rev.

[CIT0023] Barthos R, Méhn D, Demortier A (2005). Functionalization of single-walled carbon nanotubes by using alkyl-halides. Carbon N. Y.

[CIT0024] Wang Z, Yang X, Wang Q (2011). Epoxy resin nanocomposites reinforced with ionized liquid stabilized carbon nanotubes. Int. J. Smart Nano Mater.

[CIT0025] Gibson J, McKee J, Freihofer G (2013). Enhancement in ballistic performance of composite hard armor through carbon nanotubes. Int. J. Smart Nano Mater.

[CIT0026] Andreoli E, Barron AR (2015). Effect of spray-drying and cryo-milling on the CO2 absorption performance of C60 cross-linked polyethyleneimine. J. Mater. Chem. A.

[CIT0027] Dillon EP, Crouse CA, Barron AR (2008). Synthesis, characterization, and carbon dioxide adsorption of covalently attached polyethyleneimine-functionalized single-wall carbon nanotubes. ACS Nano.

[CIT0028] Zhou J, Wang C, Qian Z (2012). Highly efficient fluorescent multi-walled carbon nanotubes functionalized with diamines and amides. J. Mater. Chem.

[CIT0029] Takeuchi Y, Fujiki K, Tsubokawa N (1998). Preparation of amphiphilic carbon black by postgrafting of polyethyleneimine to grafted polymer chains on the surface. Polym. Bull.

[CIT0030] Hu H, Ni Y, Mandal SK (2005). Polyethyleneimine functionalized single-walled carbon nanotubes as a substrate for neuronal growth. J. Phys. Chem. B.

[CIT0031] Liu Y, Wu DC, Zhang WD (2005). Polyethylenimine-grafted multiwalled carbon nanotubes for secure noncovalent immobilization and efficient delivery of DNA. Angew. Chemie Int. Ed.

[CIT0032] Dong H, Ding L, Yan F (2011). The use of polyethylenimine-grafted graphene nanoribbon for cellular delivery of locked nucleic acid modified molecular beacon for recognition of microRNA. Biomaterials.

[CIT0033] Chen B, Liu M, Zhang L (2011). Polyethylenimine-functionalized graphene oxide as an efficient gene delivery vector. J. Mater. Chem.

[CIT0034] Cai X, Lin M, Tan S (2012). The use of polyethyleneimine-modified reduced graphene oxide as a substrate for silver nanoparticles to produce a material with lower cytotoxicity and long-term antibacterial activity. Carbon N. Y.

[CIT0035] Jia H, Lian Y, Ishitsuka M (2016). Centrifugal purification of chemically modified single-walled carbon nanotubes. Sci. Technol. Adv. Mater.

[CIT0036] Peñas-Sanjuán A, López-Garzón R, Domingo-García M (2012). An efficient procedure to bond nanostructured nitrogen functionalities to carbon surfaces. Carbon N. Y.

[CIT0037] Rivas BL, Geckeler KE, Abe A, Albertsson AC, Coates GW (1992). Synthesis and metal complexation of poly(ethyleneimine) and derivatives. Polymer synthesis oxidation processes.

[CIT0038] Peñas-Sanjuán A, López-Garzón R, López-Garzón J (2012). Preparation of a poly-alkylamine surface-functionalized carbon with excellent performance as a Pd(II) scavenger. Carbon N. Y.

[CIT0039] Kobayashi S, Hiroishi K, Tokunoh M (1987). Chelating properties of linear and branched poly(ethylenimines). Macromolecules.

[CIT0040] Morales-Lara F, Pérez-Mendoza MJ, Altmajer-Vaz D (2013). Functionalization of multiwall carbon nanotubes by ozone at basic pH. Comparison with oxygen plasma and ozone in gas phase. J. Phys. Chem. C.

[CIT0041] Fontanelli M, Micheloni M, International Group of Thermodynamic of Metal Complexes (1990). Potentiometric and spectrophotometric automatic titrations I Spanish-Italian Congress on Thermodynamics of Metal Complexes.

[CIT0042] Wenker H (1935). The preparation of ethylene imine from monoethanolamine. J. Am. Chem. Soc.

[CIT0043] Jones GD, Langsjoen A, Neumann SMMC (1944). The polymerization of ethyleneimine. J. Org. Chem.

[CIT0044] Hawker CJ, Lee R, Frechet JMJ (1991). One-step synthesis of hyperbranched dendritic polyesters. J. Am. Chem. Soc.

[CIT0045] Ottenbourgs BT, Adriaensens PJ, Reekmans BJ (1995). Development and optimization of fast quantitative carbon-13 NMR characterization methods of novolac resins. Ind. Eng. Chem. Res.

[CIT0046] Pierre TS, Geckle M (1985). Carbon-13 NMR analysis of branched polyethyleneimine. J. Macromol. Sci. Chem.

[CIT0047] Krämer M, Stumbé JF, Grimm G (2004). Dendritic polyamines: simple access to new materials with defined treelike structures for application in nonviral gene delivery. ChemBioChem.

[CIT0048] Krämer M, Pérignon N, Haag R (2005). Water-soluble dendritic architectures with carbohydrate shells for the templation and stabilization of catalytically active metal nanoparticles. Macromolecules.

[CIT0049] Basiuk EV, Basiuk VA, Meza-Laguna V (2012). Solvent-free covalent functionalization of multi-walled carbon nanotubes and nanodiamond with diamines: looking for cross-linking effects. Appl. Surf. Sci..

[CIT0050] Lin T, De Zhang W, Huang J (2005). A DFT study of the amination of fullerenes and carbon nanotubes: reactivity and curvature. Phys. Org. Chem.

[CIT0051] Contreras-Torres FF, Basiuk EV, Basiuk VA (2012). Nanostructured diamine-fullerene derivatives: computational density functional theory study and experimental evidence for their formation via gas-phase functionalization. J. Phys. Chem. A.

[CIT0052] Ramirez-Calera IJ, Meza-Laguna V, Gromovoy TY (2015). Solvent-free functionalization of fullerene C60 and pristine multi-walled carbon nanotubes with aromatic amines. Appl. Surf. Sci..

[CIT0053] Liao K-S, Wan A, Batteas JD (2008). Superhydrophobic surfaces formed using layer-by-layer self-assembly with aminated multiwall carbon nanotubes. Surf. Chem. Colloids.

[CIT0054] Foillard S, Zuber G, Doris E (2011). Polyethylenimine-carbon nanotube nanohybrids for siRNA-mediated gene silencing at cellular level. Pharmaceuticals.

[CIT0055] Louette P, Bodino F, Pireaux J-J (2006). Nylon 6,6 (N66) XPS reference core level and energy loss spectra. Surf. Sci. Spectra.

[CIT0056] Eby DM, Artyushkova K, Paravastu AK (2012). Probing the molecular structure of antimicrobial peptide-mediated silica condensation using X-ray photoelectron spectroscopy. J. Mater. Chem.

[CIT0057] Stevens JS, Byard SJ, Seaton CC (2014). Proton transfer and hydrogen bonding in the organic solid state: a combined XRD/XPS/ssNMR study of 17 organic acid-base complexes. Phys. Chem. Chem. Phys.

[CIT0058] Zhang L, Chatterjee A, Ebrahimi M (2009). Hydrogen-bond mediated transitional adlayer of glycine on Si(111)7×7 at room temperature. J. Chem. Phys.

[CIT0059] Yu X, Hantsche H (1993). Some aspects of the charging effect in monochromatized focused XPS. Fresenius. J. Anal. Chem.

[CIT0060] Kundu S, Wang Y, Xia W (2008). Thermal stability and reducibility of oxygen-containing functional groups on multiwalled carbon nanotube surfaces: a quantitative high-resolution XPS and TPD/TPR study. J. Phys. Chem. C.

[CIT0061] Chen C, Liang B, Ogino A (2009). Oxygen functionalization of multiwall carbon nanotubes by microwave-excited surface-wave plasma treatment. J. Phys. Chem. C.

[CIT0062] Chiang YC, Lin WH, Chang YC (2011). The influence of treatment duration on multi-walled carbon nanotubes functionalized by H2SO4/HNO3 oxidation. Appl. Surf. Sci.

[CIT0063] Steiner UB, Caseri WR, Suter UW (1993). Ultrathin layers of low- and high-molecular-weight imides on gold and copper. Langmuir.

