# Supplementary material for: Grafting the surface of carbon nanotubes and carbon black with the chemical properties of hyperbranched polyamines
Source: Sci Technol Adv Mater. 2016 Sep 12;17(1):541–53. doi: 10.1080/14686996.2016.1221728 (PMC5111562; doi:10.1080/14686996.2016.1221728)
Supplement: Supplementary_data.pdf [file tsta_a_1221728_sm2801.pdf]

Supplementary data

Grafting the surface of carbon nanotubes and carbon black with the chemical properties of hyperbranched polyamines

Francisco Morales-Lara[a], María Domingo-García[a], Rafael López-Garzón[b], M. Luz Godino-Salido[b], Antonio Peñas-Sanjuán[c], F. Javier López-Garzón[a], Manuel Pérez-Mendoza[a] and Manuel Melguizo[b]

[a]Dpto. de Química Inorgánica, Facultad de Ciencias, Universidad de Granada, 18071 Granada, Spain.

[b]Dpto. de Química Inorgánica y Orgánica, Facultad de Ciencias Experimentales, Universidad de Jaén, 23071 Jaén, Spain.

[c]Andaltec, Plastic Technology Center, Pol. Ind. Cañada de la Fuente, Calle Vílchez, s/n, 23600 Martos, Jaén, Spain.

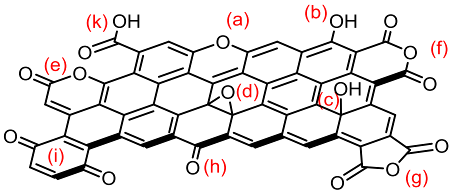

Figure S1. The main oxygen containing groups in carbon materials.

Table S1. Reactivity of the oxygen containing groups.

**Functional group**	**Reactivity with amine groups**
(a) Ether (pyrone)	No reaction
(b) Phenol	No reaction
(c) Hydroxyl	No reaction
(d) Epoxide	No reaction[a]
(e) Lactone	No reaction
(f) Cyclic anhydride (6 members)	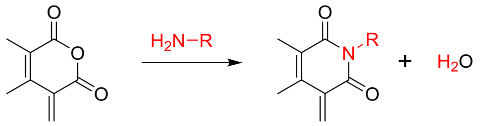
(g) Cyclic anhydride (5 members)	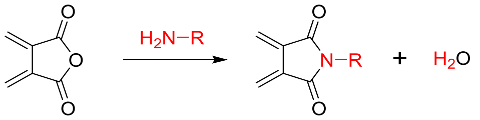
(h) Carbonyl	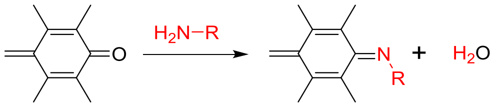
(i) Quinone	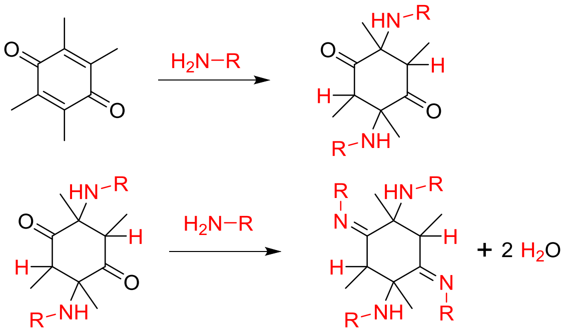
(k) Carboxylic acid	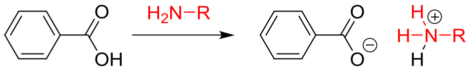

[a]Epoxides usually react with amine groups, but in carbon materials there are restrictions which hinder the reaction (see the introduction of the manuscript).

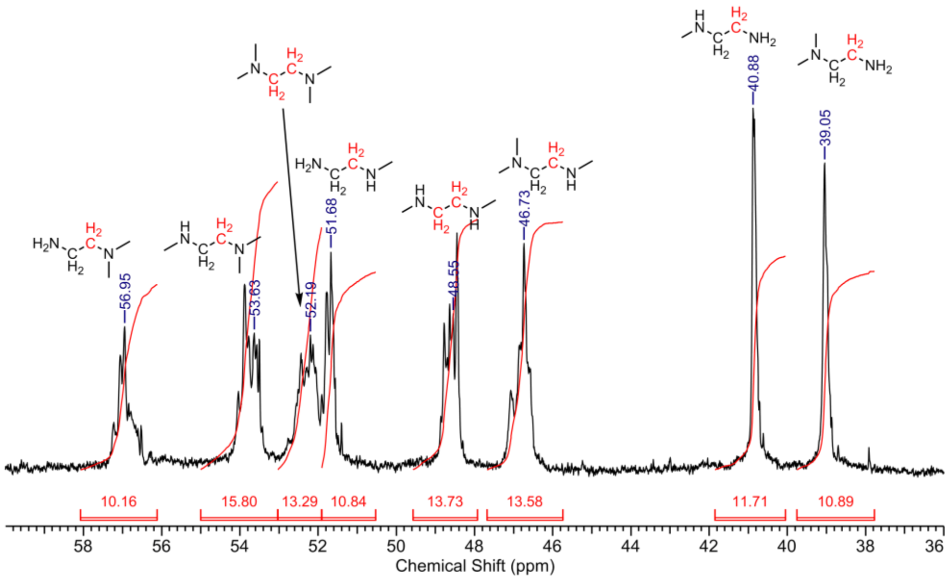

Figure S2. Quantitative ^{13}C-RMN ($CDCl_3$, 100 MHz) spectrum of $HBPEI_{600}$.

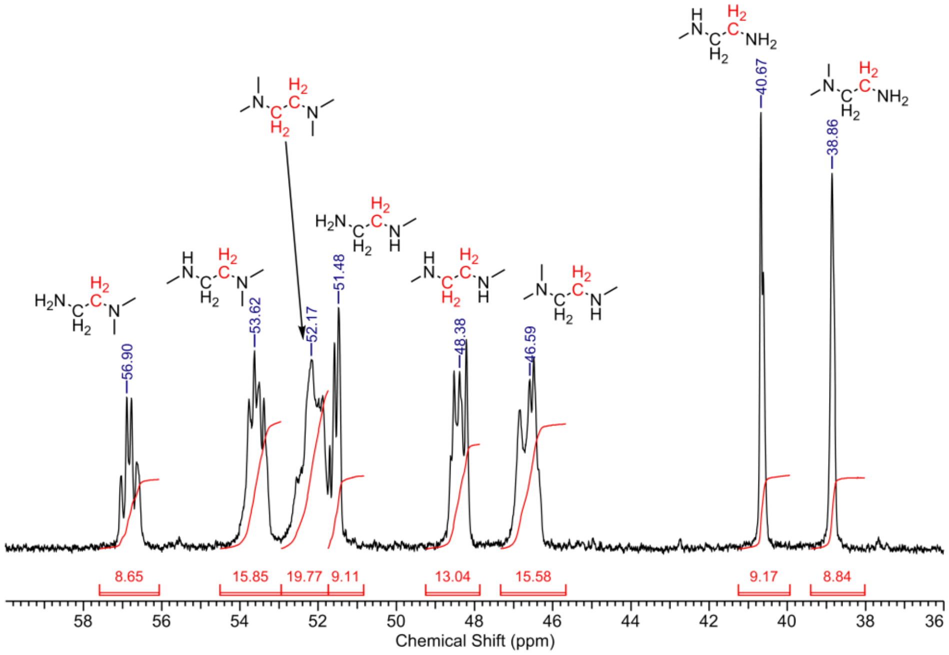

Figure S3. Quantitative ^{13}C-RMN ($CDCl_3$, 100 MHz) spectrum of $HBPEI_{1800}$.

Table S2. Chemical composition from elemental analysis.

E. A.	**C**	**N**	**O**	**H**	
MW/pH9-1800	84,7	2,6	11,8	0,9	
MW/O3G(60)-1800	89,2	3,7	5,8	1,3	
MW/OP(2)-1800	92,3	2,8	3,8	1,2	
MW/OP(30)-1800	90,6	3,9	3,9	1,6	
MW/pH9-Est-1800	92,5	2,7	3,9	1,0	
MW/OP(2)-Est-1800	91,9	2,7	4,4	1,0	
MW/OP(30)-Est-1800	90,2	3,2	5,2	1,3	
MW/pH9-600	93,2	1,6	4,5	0,7	
MW/OP(30)-600	92,9	2,7	3,4	1,0	
MW/pH9-Est-600	92,2	1,7	5,2	0,9	
	C	N	O	S	H
CSX/pH9-1800	94,7	0,6	3,4	0,7	0,6
CSX/O3G-1800	95,0	0,8	2,8	0,7	0,7
CSX/OP(2)-1800	94,6	0,7	3,4	0,8	0,7
CSX/OP(10)-1800	94,0	0,8	3,7	0,8	0,7
CSX/pH9-Est-1800	94,8	0,6	3,4	0,8	0,5
CSX/O3G(60)-Est-1800	94,5	0,6	3,6	0,7	0,5
CSX/OP(2)-Est-1800	94,4	0,6	3,7	0,8	0,5
CSX/OP(10)-Est-1800	94,1	0,7	3,9	0,8	0,5

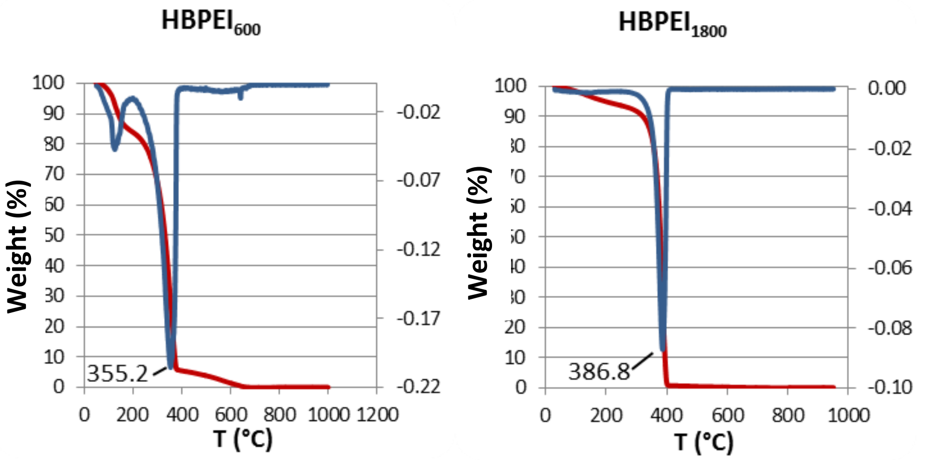

Figure S4. TG (red line) and DTG (blue line) plots of both polyamines. Experimental conditions: nitrogen flow (80 ml · min^{-1}), heating rate 10 °C · min^{-1}.

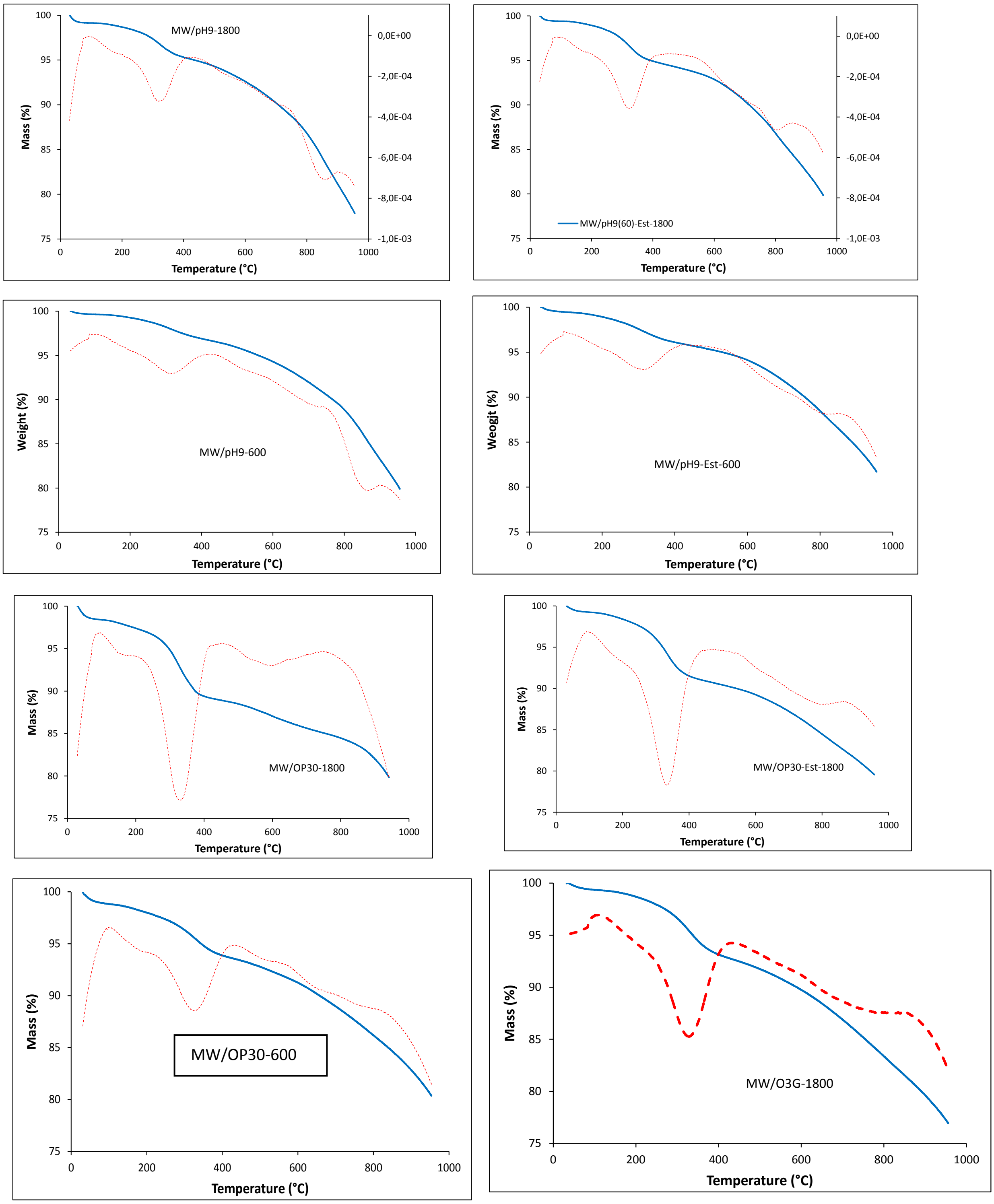

Figure S5. TG (red line) and DTG (blue line) plots of hybrids of MW-series. Experimental conditions: nitrogen flow (80 ml · min^{-1}), heating rate 10 °C · min^{-1}.

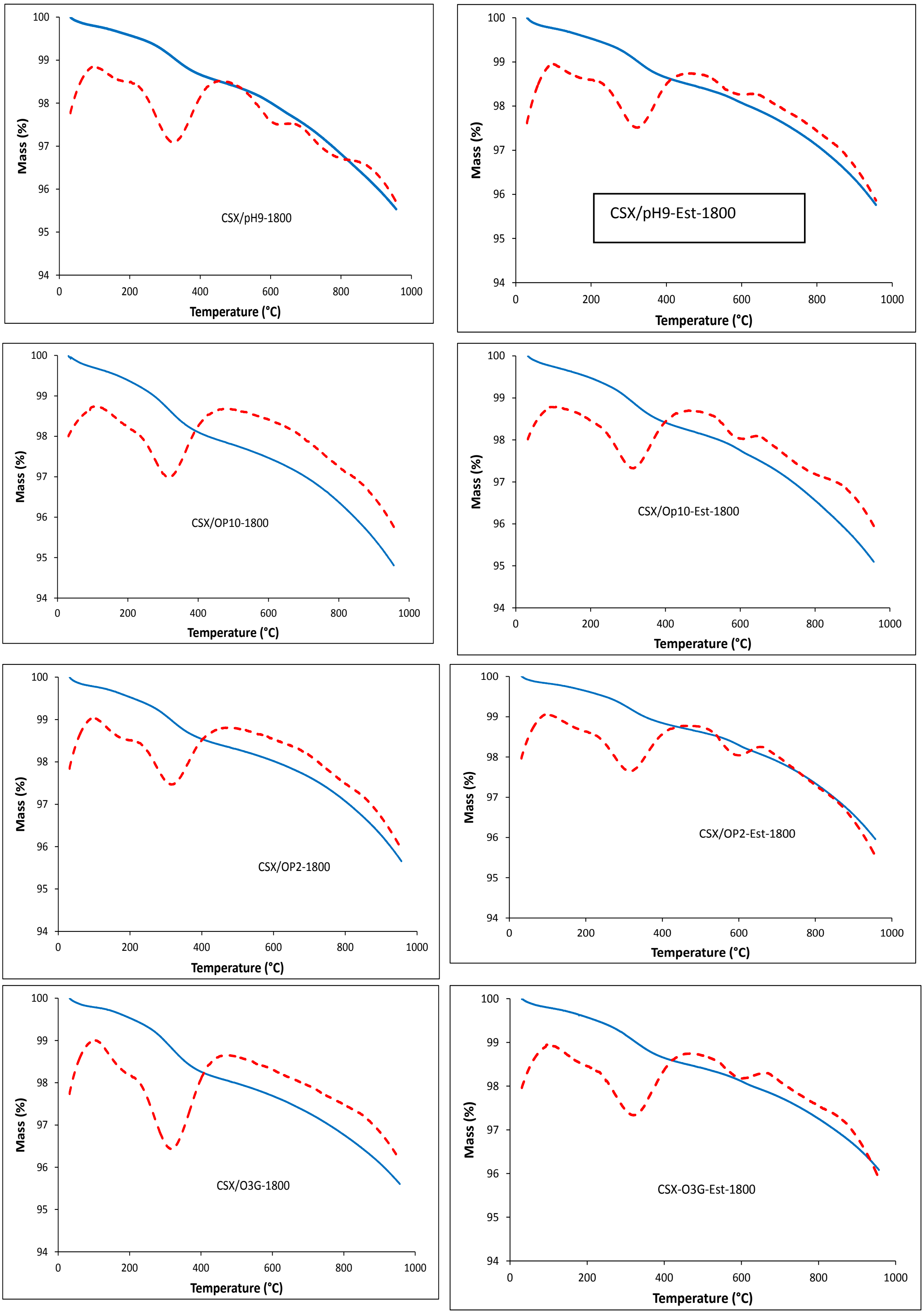

Figure S6. TG (red line) and DTG (blue line) plots of hybrids of CSX-series. Experimental conditions: nitrogen flow (80 ml · min^{-1}), heating rate 10 °C · min^{-1}.

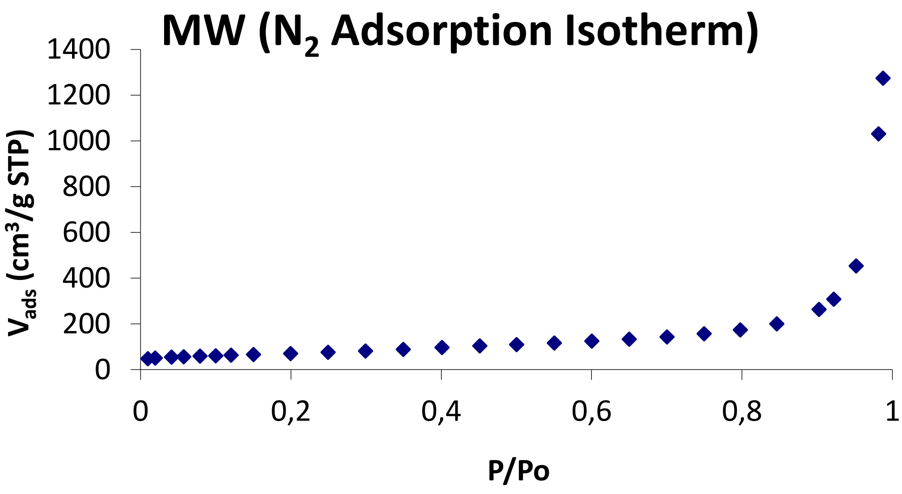

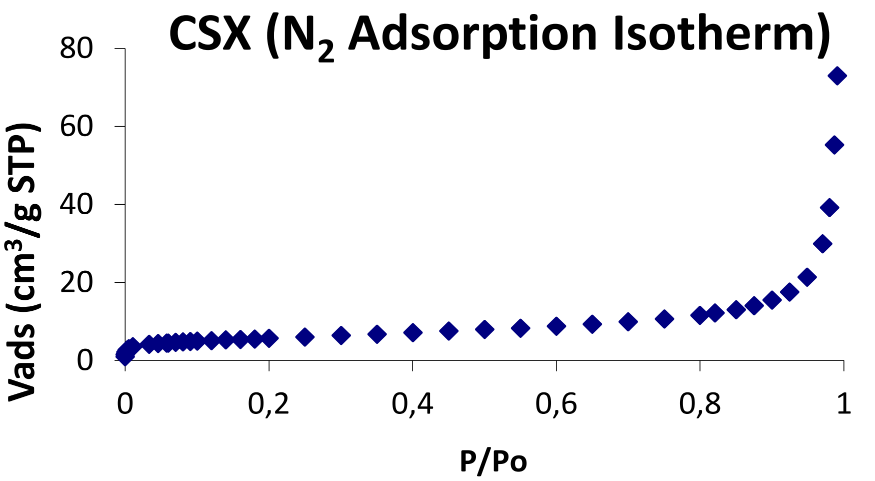

Figure S7. Nitrogen adsorption isotherms of the original MW and CSX.

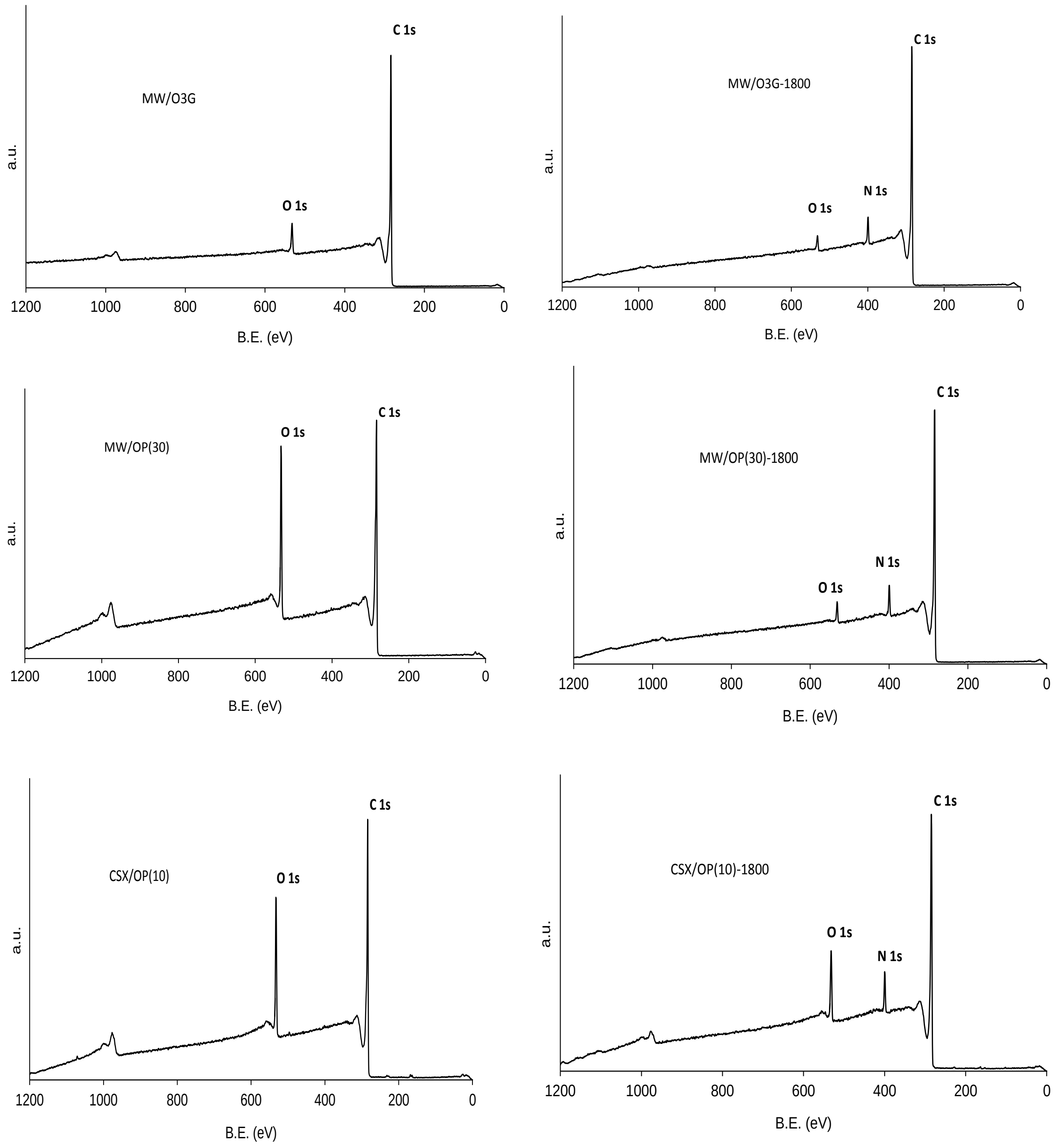

Figure S8. The XPS survey spectra of samples discussed in figure 7 of the manuscript.

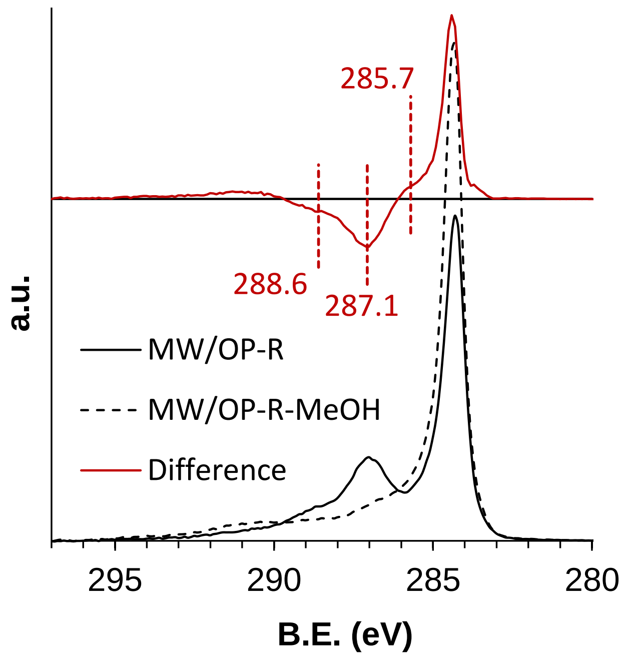

Figure S9. The treatment of a plasma oxidized sample with methanol at room temperature for 1 hour results in the loss of the labile oxygen containing groups as it seen by comparing the XPS spectra of the parent sample, MW/OP-R, and of methanol treated sample, MW/OP-R-MeOH. Both spectra and the difference are collected in this figure. Thus a significant decrease of the XPS signal in the 286-287 eV range is apparent after the treatment with methanol.

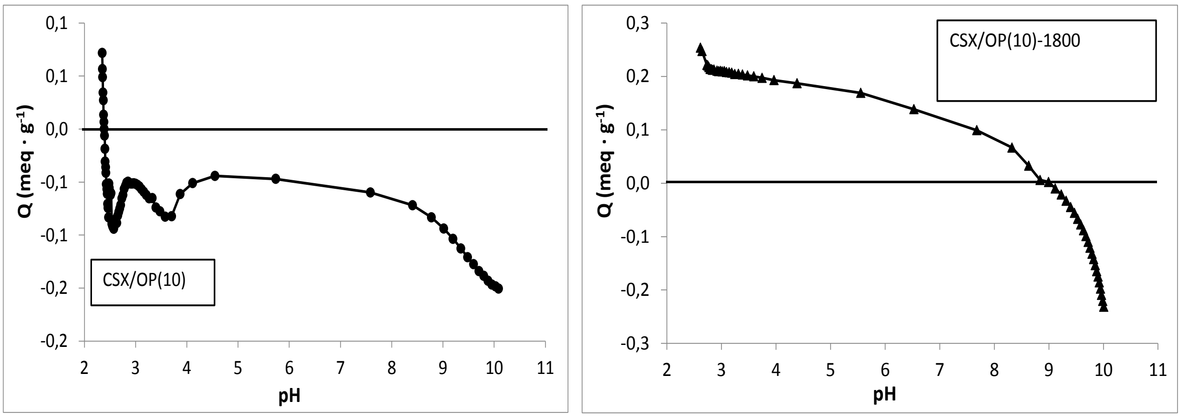

Figure S10. Plots to determine the zero point of charge.

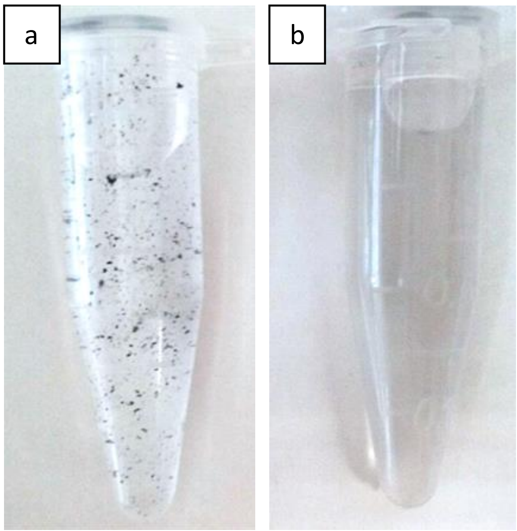

Figure S11. a) original MW and b) MW/OP(30)-1800, both in water.

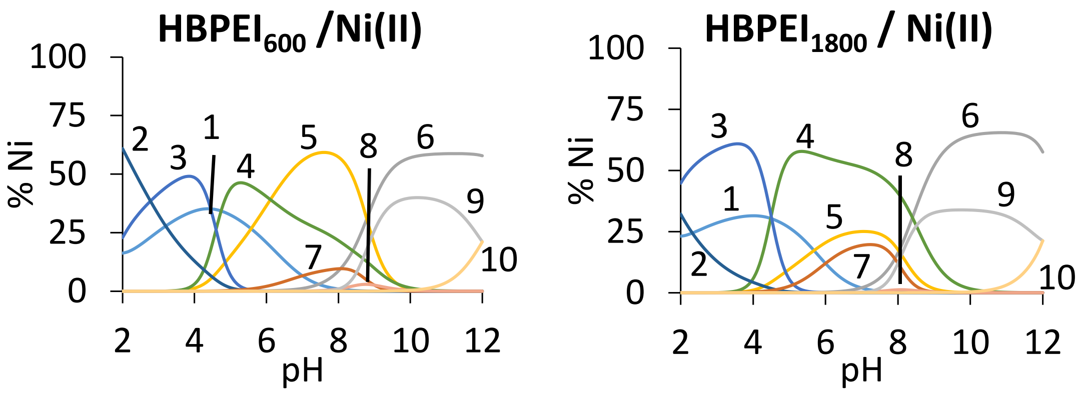

Figure S12. Species distribution plots resulting from the reaction of the polyamines with Ni^{2+}. The numbers refer to the equilibria in Table S2.

Table S3.Equilibrium constants (log K) of HBPEI/Ni(II).

	**Equilibrium**	**log K**	
		HBPEI$_{600}$	***HBPEI$_{1800}$***
1	$[H_2L]^{2+} + Ni^{2+} \leftrightarrow [NiH_2L]^{4+}$	3.19 ± 0.04	2.74 ± 0.04
2	$[H_3L_2]^{3+} + Ni^{2+} \leftrightarrow [NiH_3L_2]^{5+}$	6.66 ± 0.05	6.30 ± 0.05
3	$L + Ni^{2+} \leftrightarrow [NiL]^{2+}$	10.48 ± 0.06	10.42 ± 0.06
4	$2L + Ni^{2+} \leftrightarrow [NiL_2]^{2+}$	18.40 ± 0.05	18.60 ± 0.05
5	$[L_2Ni]^{2+} + H^{+} \leftrightarrow [NiHL_2]^{3+}$	8.40 ± 0.02	8.40 ± 0.02
6	$Ni^{2+} + OH^{-} \leftrightarrow [Ni(OH)]^{+}$	-7.00 ± 0.08	-7.00 ± 0.08
7	$[Ni(OH)]^{+} + OH^{-} \leftrightarrow [Ni(OH)_2]$	-9.00 ± 0.04	-9.00 ± 0.04
8	$[Ni(OH)_2] + OH^{-} \leftrightarrow [Ni(OH)_3]^{-}$	-7.00 ± 0.02	-7.00 ± 0.02
9	$[Ni(OH)_3]^{-} + OH^{-} \leftrightarrow [Ni(OH)_4]^{2-}$	-12.00 ± 0.02	-12.00 ± 0.02

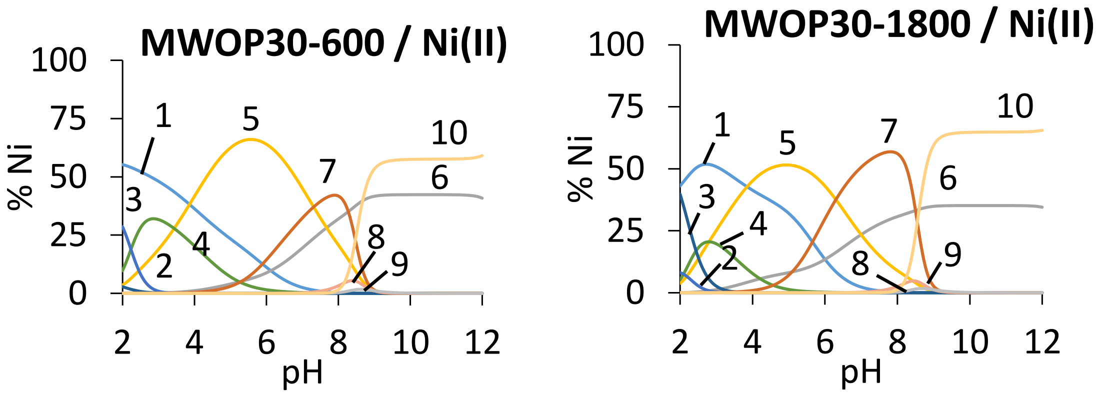

Figure S13. Species distribution plots resulting from the reaction of the hybrids with Ni^{2+}. The numbers refer to the equilibria in Table 3.

Table S4. Equilibrium constants (log K) of hybrids/Ni(II)

**Equilibrium**	**log K**	
	MWOP30-600	***MWOP30-1800***
$[H_2L]^{2+} + Ni^{2+} \leftrightarrow [NiH_2L]^{4+}$	1.84 ± 0.04	4.50 ± 0.02
$[H_3L_2]^{3+} + Ni^{2+} \leftrightarrow [NiH_3L_2]^{5+}$	5.97 ± 0.06	7.16 ± 0.08
$L + Ni^{2+} \leftrightarrow [NiL]^{2+}$	11.95 ± 0.03	11.95 ± 0.04
$2L + Ni^{2+} \leftrightarrow [NiL_2]^{2+}$	22.28 ± 0.05	22.90 ± 0.05
$[L_2Ni]^{2+} + H^{+} \leftrightarrow [NiHL_2]^{3+}$	5.25 ± 0.04	4.20 ± 0.04
$Ni^{2+} + OH^{-} \leftrightarrow [Ni(OH)]^{+}$	-5.89 ± 0.01	-5.65 ± 0.01
$[Ni(OH)]^{+} + OH^{-} \leftrightarrow [Ni(OH)_2]$	-9.11 ± 0.06	-9.35 ± 0.07
$[Ni(OH)_2] + OH^{-} \leftrightarrow [Ni(OH)_3]^{-}$	-9.00 ± 0.05	-9.00 ± 0.04
$[Ni(OH)_3]^{-} + OH^{-} \leftrightarrow [Ni(OH)_4]^{2-}$	-7.31 ± 0.01	-7.30 ± 0.01

The effective complexation constants have been determined by using the equation:

$$K_{eff} = \frac{\sum[MH_iL_j]}{\sum[H_iL_j]\sum[M]}$$

Where [M], $[H_iL_j]$ and $[MH_iL_j]$ are the metal, ligand species and abduct formed by M and H_iL_j at the equilibrium, respectively.

Table S5. A summary of the reported amount of Ni(II) captured by carbon based adsorbents.

Adsorbent	Ni(II) (mg/g)	Ref.
Activated carbón	17.8	1
Oxygen-Functionalized Carbons	72	2
Carbon-based adsorber resin Lewatit AF 5	5	3
sunflower plant biomass-based carbons	25.2	4
Activated Carbon	9.4	5
expanded graphite modified with phosphoric acid and glucose	10	6
activated carbon	10	7
Citrus limettioides Seed and **Its Carbon Derivative**	36	8
activated carbons	97.3	9
activated carbon	158.8	10
activated carbon with tetraethylenepentamine	128	11
on 1-(2-pyridylazo)-2-naphthol impregnated activated carbon cloth	43.2	12
activated carbon	48.5	13
activated carbon	166.7	14
activated carbons	40.1	15
amino functionalized magnetic graphenes	22.1	16
activated carbons	51	17
Activated carbon	17.9	18
coconut charcoal	1.8	19
Activated carbon	15	20
Activated carbon	22.2	21
Carbon black	86.9	22
Activated carbon	53.2	23
activated carbon	6.6	24
Commercial Carbons Modified with Egg Shell Wastes	9.8	25
Active carbon	31.5	26
of Coconut Oilcake Carbon	556	27
activated carbon derived from walnut shell	15.3	28
an active carbon/functionalized polyamine hybrid material	2.9	29
Pyrolytic Chars	25	30
Activated carbon	31.1	31
Activated carbon	17.4	32
activated carbon/L-lysine derivative	2.9	33
carbon nanotubes	47.8	34
chemically activated carbons	23	35
activated carbon	4.9	36
Activated carbon	54.5	37
activated carbon	62.5	38

References.

1. Rajesh, Yennam et al. DESALINATION AND WATER TREATMENT 57, 29 , 13807-13817, 2016.

2. Kern, Andreas M et al. CHEMICAL ENGINEERING & TECHNOLOGY , 39, 4 , 715-722 2016.
3. Wolowicz, Anna et al. MICROPOROUS AND MESOPOROUS MATERIALS 224 , 400-414 2016.
4. Jain, M. et al. INTERNATIONAL JOURNAL OF ENVIRONMENTAL SCIENCE AND TECHNOLOGY 13, 2, 493-500, 2016.
5. Kavand, Mohammad et ál. CHEMICAL ENGINEERING COMMUNICATIONS 203, 1, 123-135, 2016.
6. Jiang, Li et al. APPLIED SURFACE SCIENCE 357, 2355-2363, B, 2015.
7. Dwivedi, Amarendra Dhar et al. CHEMICAL ENGINEERING JOURNAL 281, 713-723, 2015.
8. Ramasamy, Sudha et al. ENVIRONMENTAL PROGRESS & SUSTAINABLE ENERGY 34, 5, 1384-1395, 2015.
9. Rao, C. Narasimha et al. DESALINATION AND WATER TREATMENT 54, 12, 3392-3399, 2015.
10. : Kalaivani, S. S. et al. DESALINATION AND WATER TREATMENT 54 , 6 , 1629-1641, 2015.
11. Ghasemi, Maryam et al. POWDER TECHNOLOGY, 274 , 362-371, 2015.
12. Alothman, Zeid A. et al. ECOTOXICOLOGY AND ENVIRONMENTAL SAFETY 112, 74-79, 2015.
13. Liu, Hai et al. RSC ADVANCES 5, 64, 52048-52056, 2015.
14. Mohammadi, Sayed Zia et al. JOURNAL OF INDUSTRIAL AND ENGINEERING CHEMISTRY 20, 6, 4112-4118, 2014.
15. Wang, Jing et al. APPLIED SURFACE SCIENCE 316, 443-450, 2014.
16. Guo, Xiaoyao et al. JOURNAL OF HAZARDOUS MATERIALS 278, 211-220, 2014.
17. Rodriguez-Estupinan, Paola et al. APPLIED SURFACE SCIENCE 286, 351-357, 2013.
18. Gao, Qun et al. POWDER TECHNOLOGY, 249, 234-240, 2013.
19. Wu, Yunhai et al. ENVIRONMENTAL SCIENCE AND POLLUTION RESEARCH 20, 4, 2210-2219, 2013.
20. Gao, Yuan et al. CHEMICAL ENGINEERING JOURNAL 217, 345-353, 2013.
21. Bouhamed, Farah et al. FRESENIUS ENVIRONMENTAL BULLETIN 22, 12 , 3490-3500, 2013.
22. Radenovic, Ankica et al. HEMIJSKA INDUSTRIJA 67, 1, 51-58, 2013.
23. Liu, Hai et al. CHEMICAL ENGINEERING JOURNAL 209, 155-162, 2012.
24. Nabarlatz, Debora et al. JOURNAL OF ENVIRONMENTAL MANAGEMENT 97, 109-115, 2012.
25. Guijarro-Aldaco, Alejandro et al. INDUSTRIAL & ENGINEERING CHEMISTRY RESEARCH 50, 15, 9354-9362, 2011.
26. Huang, LiHui et al. DESALINATION 268, 1-3, 12-19, 2011.
27. Hema, M. et al. ASIAN JOURNAL OF CHEMISTRY 22, 4, 2965-2981, 2010.
28. Wang, Gang et al. DESALINATION AND WATER TREATMENT 16, 1-3, 282-289, 2010.
29. Luz Godino-Salido, M. et al. POLYHEDRON 28, 17, 3781-3787, 2009.
30. Oezbay, N. ENERGY SOURCES PART A-RECOVERY UTILIZATION AND ENVIRONMENTAL EFFECTS 31, 14, 1271-1279, PII 911404106, 2009.
31. Madhavakrishnan, S. et al. E-JOURNAL OF CHEMISTRY 5, 4, 761-769 2008.

32. Lata, Hem et al. JOURNAL OF HAZARDOUS MATERIALS 157, 2-3, 503-509, 2008.
33. Garcia-Martin, Javier et al. EUROPEAN JOURNAL OF INORGANIC CHEMISTRY 7, 1095-1106, 2008.
34. Rao, Gadupudi Purnachadra et al. SEPARATION AND PURIFICATION TECHNOLOGY 58, 1, 224-231 2007.
35. Basso, MC et al. SEPARATION SCIENCE AND TECHNOLOGY 41, 1, 149-165. 2006.
36. Hasar, H. JOURNAL OF HAZARDOUS MATERIALS 97, 1-3, 49-57, PII S0304-3894(02)00237-6, 2003.
37. Kadirvelu, K et al. BIORESOURCE TECHNOLOGY 81, 1, 87-90, 2002.
38. Kadirvelu, K et a. SEPARATION AND PURIFICATION TECHNOLOGY 24, 3, 497-505, 2001.

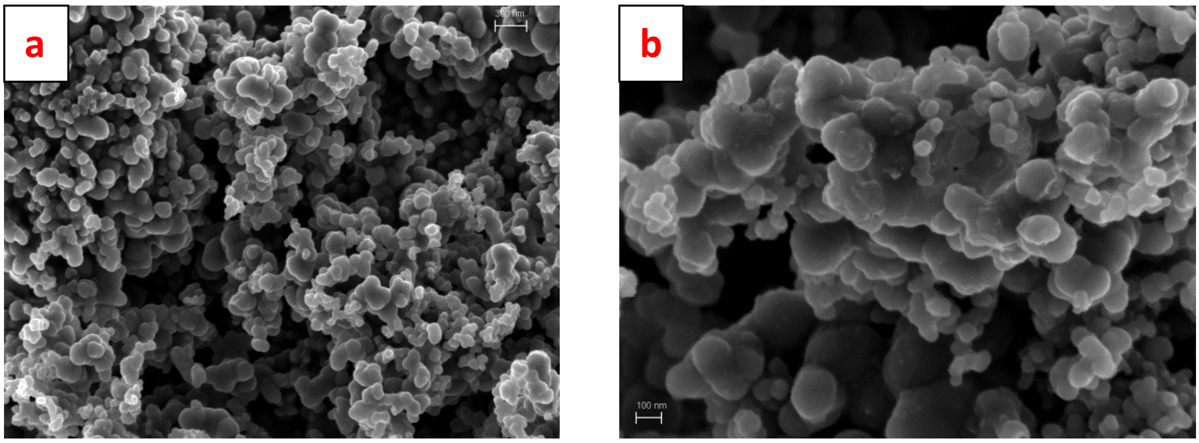

Figure S14. HRTEM images of: a) CSX/OP(10) and b) CSX/OP(10)-1800.
